# Mosaic environment-driven evolution of the deep-sea mussel *Gigantidas platifrons* bacterial endosymbiont

**DOI:** 10.1186/s40168-023-01695-8

**Published:** 2023-11-16

**Authors:** Yan Sun, Minxiao Wang, Lei Cao, Inge Seim, Li Zhou, Jianwei Chen, Hao Wang, Zhaoshan Zhong, Hao Chen, Lulu Fu, Mengna Li, Chaolun Li, Song Sun

**Affiliations:** 1 CAS Key Laboratory of Marine Ecology and Environmental Sciences, and Center of Deep Sea Research, Institute of Oceanology, Chinese Academy of Sciences, Qingdao, 266071 China; 2 Laboratory for Marine Ecology and Environmental Science, Laoshan Laboratory, Qingdao, 266237 China; 3 Integrative Biology Laboratory, College of Life Sciences, Nanjing Normal University, Nanjing, 210046 China; 4 School of Biology and Environmental Science, Queensland University of Technology, Brisbane, QLD 4000 Australia; 5 BGI Research-Qingdao, BGI, Qingdao, 266555 China; 6 South China Sea Institute of Oceanology, Chinese Academy of Sciences, Guangzhou, 510301 China; 7 University of Chinese Academy of Sciences, Beijing, 100049 China

**Keywords:** Deep sea, Horizontally transmitted endosymbionts, Within-species diversity, Mobile genetic elements, Genome evolution, Environmental adaptation

## Abstract

**Background:**

The within-species diversity of symbiotic bacteria represents an important genetic resource for their environmental adaptation, especially for horizontally transmitted endosymbionts. Although strain-level intraspecies variation has recently been detected in many deep-sea endosymbionts, their ecological role in environmental adaptation, their genome evolution pattern under heterogeneous geochemical environments, and the underlying molecular forces remain unclear.

**Results:**

Here, we conducted a fine-scale metagenomic analysis of the deep-sea mussel *Gigantidas platifrons* bacterial endosymbiont collected from distinct habitats: hydrothermal vent and methane seep. Endosymbiont genomes were assembled using a pipeline that distinguishes within-species variation and revealed highly heterogeneous compositions in mussels from different habitats. Phylogenetic analysis separated the assemblies into three distinct environment-linked clades. Their functional differentiation follows a mosaic evolutionary pattern. Core genes, essential for central metabolic function and symbiosis, were conserved across all clades. Clade-specific genes associated with heavy metal resistance, pH homeostasis, and nitrate utilization exhibited signals of accelerated evolution. Notably, transposable elements and plasmids contributed to the genetic reshuffling of the symbiont genomes and likely accelerated adaptive evolution through pseudogenization and the introduction of new genes.

**Conclusions:**

The current study uncovers the environment-driven evolution of deep-sea symbionts mediated by mobile genetic elements. Its findings highlight a potentially common and critical role of within-species diversity in animal-microbiome symbioses.

Video Abstract

**Supplementary Information:**

The online version contains supplementary material available at 10.1186/s40168-023-01695-8.

## Background

Symbiotic relationships between microbes and animals are ubiquitous in nature. Symbionts shape the ecology and evolution of animal hosts and contribute to the immense present-day diversity in animal lifestyles [1]. Microbial genomes encode a wide range of functions, facilitating the development of radically novel phenotypes and adaptive features during their symbiosis with the animal host. By developing new metabolic or physiological capabilities, symbionts help their host exploit new ecological niches [2–4]. Many animals inhabiting the deep sea have adapted to their challenging environment by establishing symbiotic relationships with microbes. Diverse mutualistic relationships between deep-sea animals and their prokaryotic symbionts have been established over evolutionary time, enabling holobionts to utilize a variety of energy resources to colonize a wide range of habitats in deep-sea hydrothermal vent and methane seep ecosystems worldwide [5].

Within-species variation of symbionts brings ecological and evolutionary advantages that benefit an obligate symbiosis relationship. Such advantages include differential utilization of diverse environmental substrates and increased potential for adaptation to new and changing environments [6–8]. For example, recent studies revealed that multiple strains or subspecies of a single bacteria may occur concurrently within the same or different host individuals [9]. Although the genome assembly of highly similar bacteria remains challenging, population genetics analyses based on nucleotide sequences have revealed pervasive within-species diversity in many deep-sea endosymbionts [7, 8, 10–13]. Moreover, complex metagenomic gene coverage or content analyses have identified substantial functional heterogeneity, even within a single animal host. For instance, as many as 16 strains of intracellular sulfur-oxidizing bacterial symbionts were shown to coexist in a *Bathymodiolus* mussel [8]. These strains differed markedly with respect to many key functions, including energy and nutrient sources, electron acceptors, and viral defense mechanisms. Such functional differences may support the local adaptation of the host by conferring metabolic flexibility to effectively exploit resources available in highly heterogenous and fluctuating deep-sea geochemical environments [7, 8, 12].

Symbiont heterogeneity is strongly influenced by transmission mode [1]. In marine ecosystems, microbial symbionts are primarily transferred between host generations via the external environment (i.e., horizontal transmission) [12, 14]. Compared with the direct inheritance of parental symbiont lineages (i.e., vertical transmission), symbiotic relationships that rely on horizontal transmission frequently acquire potentially heterogenous symbionts from surrounding host individuals or the environment, causing high symbiont diversity. Moreover, the “free-living” period that is obligate in the horizontal transmission mode exposes the symbiont to environmental influences and selection pressures. The exposure of horizontally transmitted symbionts to the environmental gene pool also facilitates the maintenance of high functional plasticity by mobile genetic elements (MGEs), such as plasmids and transposable elements (TEs) [15]. MGEs can mediate intra- or intercellular DNA trafficking and, therefore, contribute to bacterial adaptation and evolution [16–18]. MGEs typically carry accessory cargo genes that encode important adaptive functions and have been shown to improve symbiosis in many model organisms [19, 20]. Despite their importance in adaptive evolution, our understanding of the contributions of MGEs to deep-sea symbioses, especially those involving horizontally transmitted symbionts, remains in its infancy. However, recent studies have revealed a substantial level of TE enrichment in certain deep-sea horizontally transmitted symbionts [21–24]. For example, in the bacterial symbionts of deep-sea snails, TEs represent more than 10% of total genes [25]. The functions of these enriched MGEs and their influence on bacterial functional heterogeneity and adaptive genome evolution are poorly understood.

Bathymodioline mussels are deep-sea organisms with environmentally acquired endosymbionts and are studied due to their adaptive and evolutionary plasticity [26, 27]. *Gigantidas platifrons*, endemic to the Northwest Pacific, is one of few bathymodioline species capable of inhabiting two chemically distinct ecosystems: hydrothermal vents and methane seeps [28]. Hydrothermal vents expel more toxic chemical substances, such as hydrogen sulfide (H_2_S) and heavy metals, than methane seeps [29]. Population genetic analysis of the *G. platifrons* has detected genetic divergence between the seep population in the South China Sea and other groups from the open Northwest Pacific, revealing the co-occurrence of two cryptic semi-isolated host lineages due to a genetic barrier to gene flow [30]. Based on electron microscopy observations and 16s rDNA analyses, the *G. platifrons* hosts only have a single species of methanotrophic endosymbiont, irrespective of deep-sea habitat [28, 31, 32]. Nevertheless, our previous metatranscriptomic analysis identified differences in the expression patterns of a sulfide-oxidizing gene between vent- and seep-inhabiting mussel symbionts, hinting at functional heterogeneity within the sole endosymbiont lineage. Thus, *G. platifrons* is an excellent model for studying the within-species variation of a symbiotic microbe and its contribution to environmental adaption.

Recently developed metagenome sequencing methods allow the generation of fine-scale metagenomic assemblies that can distinguish small genomic differences between closely related microbes [33]. Here, we used PacBio long-read (HiFi) and Illumina sequencing to assess within-species variation of the sole symbiont of *G. platifrons* populations from a methane seep (South China Sea) and two hydrothermal vents (Okinawa Trough). We also conducted a metagenomic comparison of TEs and plasmids among these deep-sea symbionts to clarify how MGEs mediate genome functional heterogeneity and promote genetic innovation in response to environmental conditions. Our results improve knowledge of the relationship between functional heterogeneity and environmental adaptations in horizontally transmitted endosymbionts and, more importantly, the evolutionary forces shaping symbiont-host diversification.

## Results

### Mussel collection and geochemical features of the sampling sites

**Fig. 1:**
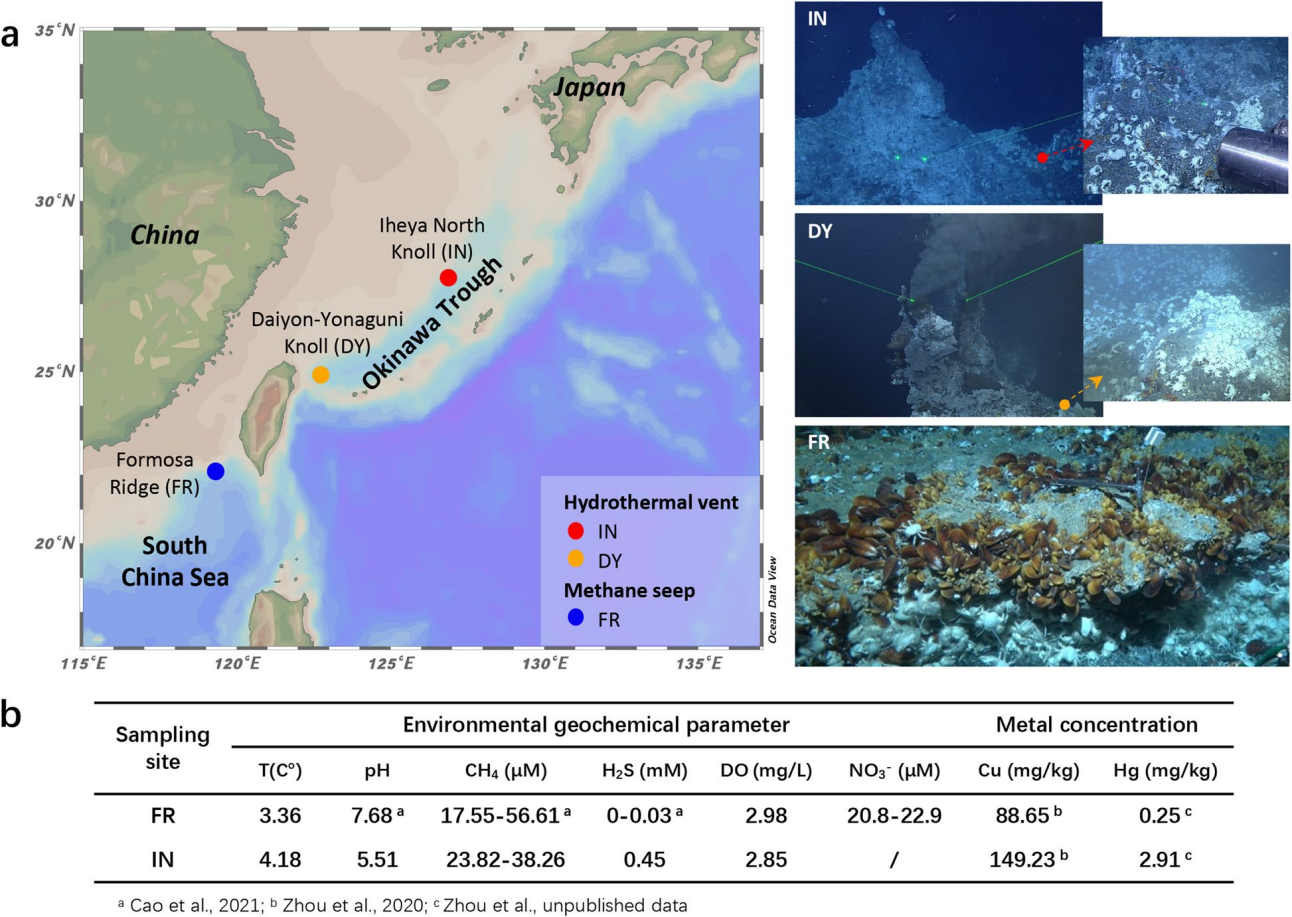
Environmental characteristics of the mussel collection sites. **a** Map depicting the locations of the methane seep and hydrothermal vent fields sampled. Photographs are representative images of each sample site, showing the *G. platifrons* and *Shinkaia crosnieri* (squat lobster) dominated communities. Map created using Ocean Data View (ODV) v.5.0 (https://odv.awi.de). **b** Summary of the main geochemical parameters of the seawater surrounding the chemosynthetic colonies at the FR and IN sites, as well as the mean concentrations of heavy metals (mg/kg dry weight) in the gill tissues of *G. platifrons*. Note that site DY is geochemically similar to site IN

The *G. platifrons* used in this study were obtained from a methane seep (hereafter seep) in the South China Sea (Formosa Ridge, FR) and two hydrothermal vents in the Okinawa Trough (middle: Iheya North Knoll, IN; southern: Daiyon-Yonaguni Knoll, DY; Fig. 1a). The *in situ* geochemical parameters of the aquatic environments surrounding the chemosynthetic *G. platifrons* colonies in site FR and site IN [methane (CH_4_), H_2_S, dissolved oxygen (DO), nitrate (NO_3_^−^), and pH] were measured in this study and in our previous study [34]. The geochemical characteristics of DY were not measured; however, previous studies [35, 36] have shown that it is geochemically similar to site IN. The results of geochemical parameters are summarized in Fig. 1b. High levels of CH_4_ were detected at site FR and site IN, likely explaining the success of *G. platifrons* at these sites due to their methane-oxidizing endosymbionts. DO was similar between the two sites but was slightly lower at IN. H_2_S levels differed markedly between the hydrothermal vent (IN) and the methane seep (FR). The NO_3_^−^ concentration at site FR was also higher than that at site IN (0 µM) [35]. The vent environment was also more acidic (i.e., lower pH) than the methane environment. Finally, our previous assessment of copper (Cu) and mercury (Hg) concentrations in mussel gill tissues showed that FR (seep) and IN (vent) were rich in these heavy metals, although heavy metal concentrations were noticeably higher at the vent site [29].

### High-resolution assembly of endosymbiont genomes reveals three phylogenetic clades

Metagenomic sequencing of the *G. platifrons* samples was performed using Illumina short reads and PacBio long reads (Supplementary Fig. 1; Supplementary Table 1). The Illumina data were analyzed by a MetaWRAP-based binning pipeline to overview the composition of gill-associated microbes. The dominant bacteria associated with the gills were all recovered with high quality (Supplementary Fig. 2). Taxonomic analysis of Illumina-produced sequences from 22 individual mussels revealed that a single phylotype of methane-oxidizing symbionts made up most of the gill-associated microbial community in both seep and vent mussels (Supplementary Note 1; Supplementary Fig. 2). The high-quality genome assemblies of the primary methanotrophic symbionts were unveiled by a newly developed reads-binning pipeline (Supplementary Fig. 1). The pipeline was able to better capture within-species genomic information compared to the traditional binning methods, as confirmed by sixteen mocked test runs of simulated sequencing reads from published *Escherichia coli* strains (eight simulated PacBio HiFi reads, binned by two sets of Illumina reads respectively; details were given in Supplementary Note 2; Supplementary Tables 2 and 3; Supplementary Figs. 3-5). Based on five PacBio HiFi sequencing datasets of *G. platifrons* (two from FR and three from IN; Supplementary Table 1), 28 bacterial genomes with average completeness of 89.21% and an average genome size of 3.53M (Supplementary Table 4) were obtained. The GC content of the assembled genomes was 40.8–41.0% (Supplementary Table 4). Assessment of the genome recovery rate was conducted using both PacBio and Illumina data, demonstrating the completeness of the assembled genomes in representing the within-species genomic diversity in both vent and seep individuals (Supplementary Note 3; Supplementary Fig. 6).

**Fig. 2:**
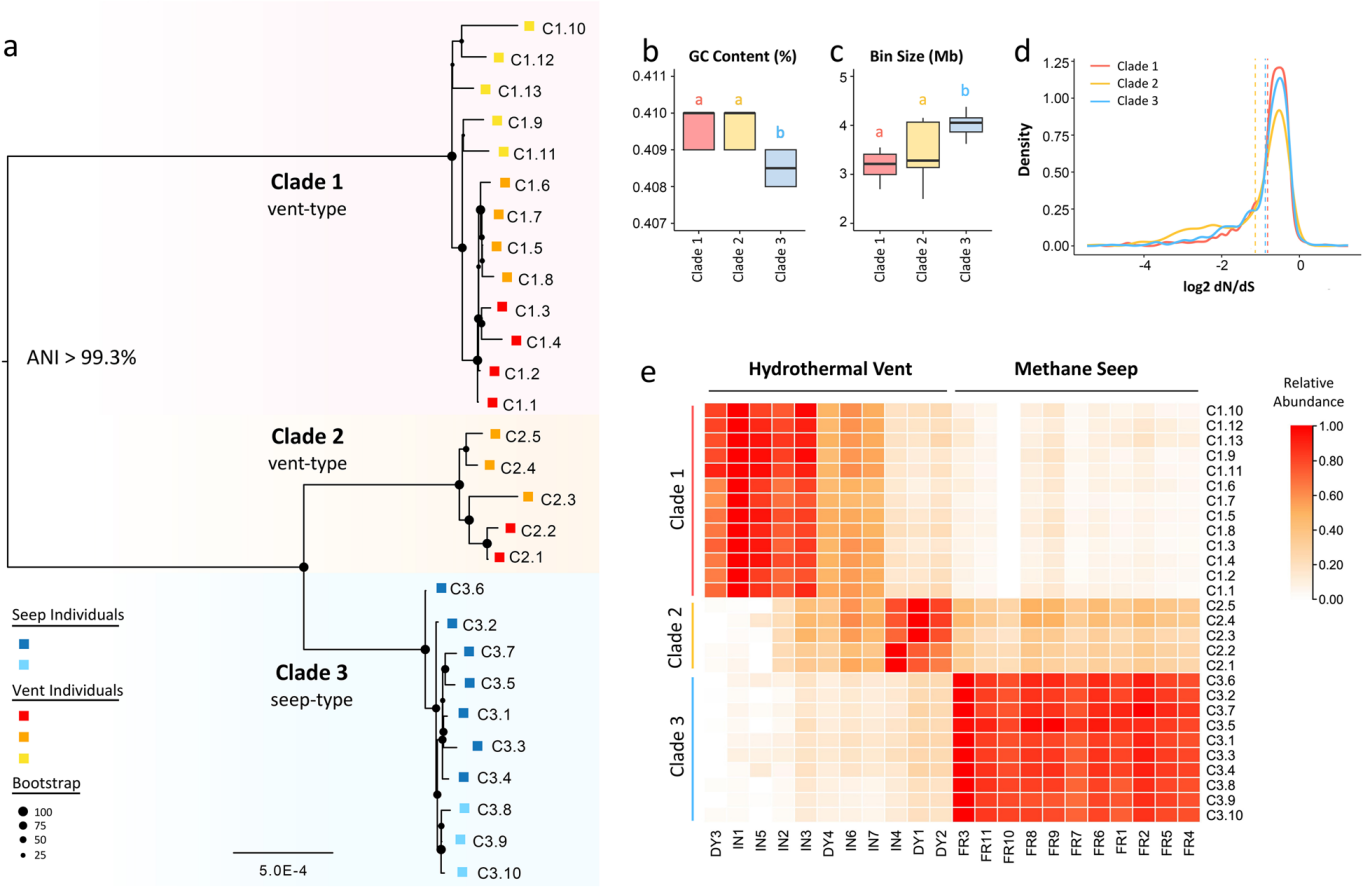
Genetic divergence of methane-oxidizing endosymbionts between vent and seep mussels. **a** ML phylogenetic reconstruction of the assembled endosymbiont genomes based on orthologous genes conserved across the pangenome. A total of five PacBio HiFi sequencing datasets were used for genome assembly. Every dataset was assembled separately, and the squares prepending each sequence label correspond to specific datasets derived from seep and vent mussel individuals. **b** GC content of the assembled genomes in the three clades. **c** Bin size of the assembled genomes in the three clades. **d** Density plot of the dN/dS values for each clade estimated from 1000 concatenated alignments constructed from twenty-five randomly chosen orthologs. Dash line showing the mean value of each clade. **e** Distribution of endosymbiont abundance across individual mussels collected from vent (Iheya North Knoll, IN, and Daiyon-Yonaguni Knoll, DY) and seep (Formosa Ridge, FR) environments

Phylogenetic reconstruction of orthologous genes conserved across the endosymbiont pangenome recovered three well-defined lineages (Fig. 2a; Supplementary Fig. 7). Notably, the cluster of the genomes followed an environment-linked pattern: clades 1 and 2 were composed of genomes assembled from the vent-associated mussels, whereas clade 3 was composed of genomes assembled from the seep-associated mussels (Fig. 2a). Thus, we considered clades 1 and 2 vent-type clades and clade 3 seep-type clades (Fig. 2a). In each clade, the genomes also showed a tendency to cluster by mussel individual, implying that some closely related genomes coexist in the same individual, proposing the possibility of intra-host evolution in symbionts.

The pairwise average nucleotide identity (ANI) values of the assembled genomes ranged from 99.30–99.98% (Supplementary Fig. 8), exceeding the 95% threshold for bacterial species definition [37]. However, assigning strains and subspecies is not straightforward [38]. Therefore, we considered them distinct within-species variants. The range of ANI values for clades 1, 2, and 3 were 99.82–99.96%, 99.85–99.98%, and 99.92–99.96%, respectively. The ANI values among clades were relatively lower and revealed different degrees of genetic divergence among the three clades: clade 1 was more dissimilar to clades 2 and 3 (ANI values range from 99.30 to 99.41%), while the latter two clades were more similar to each other (ANI values range from 99.61 to 99.68%; Supplementary Fig. 8). Clustering patterns based on genome features were consistent with the lineages recovered by phylogenetic and ANI analyses and revealed an environment-linked pattern. The average GC content of clades 1 and 2 genomes was slightly higher than that in clade 3 (Fig. 2b, Supplementary Table 4), likely reflecting the former vent-derived genomes’ greater thermal stability. The assembled genomes in clades 1 and 2 were smaller in size than those in clade 3 (Fig. 2c, Supplementary Table 4). Genome structure analysis revealed similar patterns of differentiation among the clades. Whole-genome alignments of representative genomes (i.e., the genome with the highest contig N50 value in each clade) supported the results of the phylogenetic analysis. They revealed that genomes within the same clade were highly syntenic, with fewer rearrangements, insertions, or deletions, whereas genomes in different clades were far more structurally dynamic (Supplementary Fig. 9).

The evolutionary rate of the three clades was compared by estimating the dN/dS values (ratios of nonsynonymous to synonymous substitutions) for each clade. Results obtained from orthologous genes (Supplementary Fig. 10) and 1,000 concatenated alignments constructed from twenty-five randomly chosen orthologs (Fig. 2d) revealed that clades 1 (vent) and 3 (seep) had relatively higher mean dN/dS ratios than the clade 2 (vent). Among the two vent clades, the dN/dS ratio in clade 1 was significantly higher than that of clade 2 (Wilcoxon rank-sum test, *P* < 0.01; Fig. 2d), indicating accelerated evolution of this lineage in the vent environment. Additionally, selection analysis identified positive gene selection differences between the cases associated with metabolite biosynthesis and environmental adaptation (Supplementary Note 4). These results suggest a contribution of the environment to the functional differentiation of the symbionts.

### Within-species divergence of endosymbionts between the vent and seep mussels

Phylogenetic analysis revealed three environment-linked clades, implying within-species divergence of symbionts living in different habitats (Fig. 2a). We next estimated their relative abundance across 22 mussels collected from the three sampling sites (11 from FR, seven from IN, and four from DY) based on the average mapping depth of Illumina short reads. Considering that the genome sequences were highly similar, we used strict criteria for mapping—retaining reads with less than three mismatches and only kept primary hits (either unique- or multi-match sequences) with the highest mapping scores. A heatmap revealed a distinct distribution pattern among mussel individuals from the vent and seep sites (Fig. 2e). Symbionts of the vent-associated mussels (IN and DY) were composed of vent-type clades 1 and 2, despite variations in relative abundance among individuals. Meanwhile, seep-type clade 3 was detected at high abundance in the mussels from the seep site (FR; Fig. 2e). The heatmap showed signals of clade 2 in the seep mussels; however, this may be caused by multi-matching Illumina reads aligned to the higher similarity genomes of the two clades.

To provide further evidence of our results based on genome assembly, we performed assembly-independent genome-wide SNP analyses [8, 13] to estimate the genetic divergence of symbiont populations among individual mussels from different sites (Supplementary Note 5). The pairwise fixation index (*F*_ST_) values among individual mussels based on SNPs in orthologous genes revealed habitat-associated population differentiation of individual mussels (Supplementary Fig. 11). *F*_ST_ values were much higher among vent (0.117–0.709) than that among seep (0.088–0.277) mussels, which may be due to abundance differences among the two vent clades (Supplementary Fig. 11; Supplementary Note 5). Principal coordinate analysis (PCoA) also revealed a clear population separation of the symbionts from vent and seep sites (Supplementary Fig. 12). In addition, SNPs also recovered three environment-linked clades (Supplementary Fig. 13a). Their distributions across individual mussels showed a similar pattern to the three clades detected through our metagenome assembly (Supplementary Fig. 13b).

### Function comparisons among the three clades

**Fig. 3:**
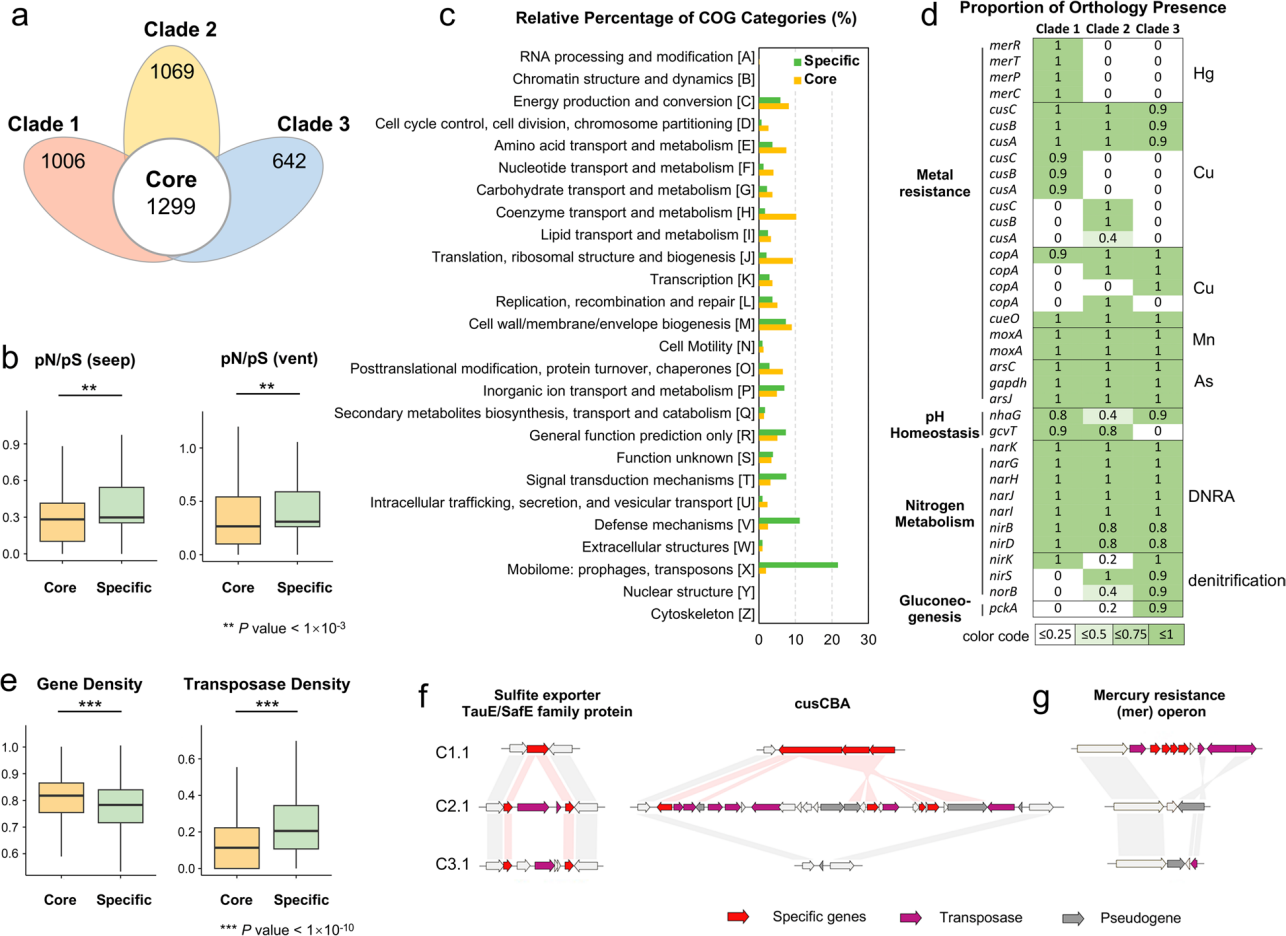
Functional characterization of the methane-oxidizing endosymbiont clades and influences of TEs on clade-specific functional differentiation. **a** Venn diagram showing numbers of orthologous genes shared and unique across the endosymbiont clades. **b** Box plot showing nonsynonymous to synonymous polymorphisms (pN/pS) in the core genes and the clade-specific genes in seep and vent populations. **c** COG terms enriched in the orthologous core and clade-specific genes. **d** Heatmap showing the percentage of environmental-related orthologs within the three clades. **e** Box plots showing gene density and transposase density in the 3-kb sequences flanking the core and clade-specific genes. **f** Synteny analysis of the scaffold regions around a clade-specific gene (here, sulfite exporter TauE/SafE family protein and the copper efflux pump *cusCBA*) in the three clades (C1.1, C2.1, and C3.1), showing that the insertion of a transposase results in the disruption, rearrangement, and non-functionalization of the corresponding gene. **g** Synteny analysis of the scaffold regions around clade-specific genes inserted by larger transposon carrying cargo genes (here, the *mer* operon)

We explored differences in gene function repertoires among clades by first identifying core (shared) and clade-specific (unique) orthologous genes. To minimize bias from incomplete genome assemblies, we defined core genes as orthologous genes shared in more than 90% of all the genomes. To identify clade-specific genes, we initially identified genes overrepresented in a particular clade using Fisher’s exact test and then intersected genes uniquely present in that clade. Across all assembled genomes, 1299 were identified as core genes among the total orthologous gene groups, while 1006, 1069, and 642 were clade-specific genes in clades 1, 2, and 3, respectively (Fig. 3a). The ratio of nonsynonymous to synonymous polymorphisms (pN/pS), a variant of dN/dS that corresponds to selection pressure, was significantly lower for core genes (0.316 ± 0.010 and 0.381 ± 0.012 in seep and vent populations, respectively) than for clade-specific genes (0.400 ± 0.015 and 0.444 ± 0.012 in seep and vent populations; *P* < 1×10^−3^), indicating that core genes are more conserved in comparison with clade-specific genes (Fig. 3b). Estimation of dN/dS values also supported the higher strength of purifying selection on core genes (Supplementary Fig. 14).

The function of core genes and clade-specific genes was compared by COG (Clusters of Orthologous Groups) functional analysis (Fig. 3c). The results showed that core orthologs were associated with central metabolic-related categories, including coenzyme, carbohydrate, amino acid, and nucleotide transport and metabolism (Fig. 3c). KEGG and GO enrichment analysis further supported that core genes were enriched in biosynthesis-related pathways and GO terms, such as biosynthesis of cofactors (ko01240), phenylalanine, tyrosine and tryptophan biosynthesis (ko00400), vitamin biosynthetic process (GO:0009110), and aromatic compound biosynthetic process (GO:0019438; Supplementary Table 5). Other conserved pathways highlighted by functional enrichment analysis included the transport of substances and compounds (Supplementary Table 5). Cofactors (e.g., vitamins and biotin) and essential amino acids (e.g., aromatic amino acids) are indispensable nutrients but cannot be synthesized by the host mussels [39, 40]. Therefore, our results suggest that the core genes of symbionts are essential for nutrient biosynthesis and transportation within the symbiotic relationship. Additionally, the core genes also encode enzymes associated with redox maintenance, ion homeostasis, and the detoxification or oxidation of common environmental metals, such as arsenate (As) and manganese (Mn) (Fig. 3d).

In contrast to the core genes, the COG functional analysis indicated that the clade-specific genes are associated with signal transduction mechanisms, defense mechanisms, and mobilome (Fig. 3c). In addition, KEGG and GO enrichment analysis also suggested that the clade-specific genes are involved in environmental adaptation (Supplementary Table 6). The clade-specific genes of the two vent clades were enriched for the two-component system (ko02020), response to chemicals (GO:0042221), and response to toxic substances (GO:0009636). These processes enable bacteria to sense, respond, and adapt to environmental changes, such as high levels of heavy metals. The clade-specific genes also included genes involved in the utilization of environmental substrates. For example, clade 3-specific genes were enriched for pathways of pyruvate metabolism (ko00620) and glycolysis/gluconeogenesis (ko00010). Other enriched pathways also included base excision repair (ko03410), glycine catabolic process (GO:0006546), and transposition (GO:0032196).

We next compared the percentage of important environmental adaptation-related orthologous genes in each clade, including genes involved in heavy metal resistance, pH homeostasis, nitrate utilization, and gluconeogenesis (Fig. 3d; Supplementary Table 7). Strikingly, the mercury (Hg) resistance (*mer*) operon was found in all the genomes in clade 1 but was absent in clades 2 and 3 (Fig. 3d). The *mer* operon, which encodes the mercuric ion (Hg^2+^)-responsive activator MerR and the Hg^2+^ transporter MerTPC, mediates the transports Hg^2+^ from the extracellular space into the bacterial cytosol. In contrast, genes involved in the copper (Cu) homeostatic regulatory systems, *cue* (comprised of Cu^+^ translocating P-type ATPase CopA and multicopper oxidase CueO) and *cus* (the copper efflux pump CusCBA), were found in all three clades, although gene copy numbers differed among clades. Finally, the glycine cleavage system T-protein (GcvT), which participates in cellular pH homeostasis by catalyzing the oxidative cleavage of glycine and producing NH_4_^+^, was only present in the two vent clades. Differences in environmental substrate utilization and metabolite biosynthesis were found among the three clades. For example, the dissimilatory nitrate reduction to ammonium (DNRA) pathway, which includes the nitrate reductase NarGHJI and nitrite reductase NirBD, was conserved across the three clades. However, the denitrification pathways in clade 1 and clade 2 were incomplete due to a complete or partial loss of related genes, including those encoding NO-generating nitrite reductase NirK/NirS and N_2_O-generating nitric oxide reductase NorB. Additionally, phosphoenolpyruvate carboxykinase (*pckA*), which encodes a protein that catalyzes the conversion of oxaloacetate (OAA) to phosphoenolpyruvate (PEP) and is, thus, critical for gluconeogenesis, was found only in clade 3. A detailed description of the functional genes identified in the three clades can be found in Supplementary Note 6.

### TEs contribute to the differentiation of the symbiont clades

The COG functional category “mobilome: prophages, transposons” was enriched in the clade-specific genes, implying that TEs potentially played an important role in the differentiation of the three symbiont clades (Fig. 3c). To explore TE function in these bacteria further, we predicted the TEs present as insertion sequences (ISs) in the orthologous gene. ISs are short TEs that contain genes coding for proteins involved in transposition (i.e., transposase). Substantially, more TEs were detected in the clade-specific genes (13.90 ± 0.21%, 11.91 ± 1.35%, and 15.14 ± 0.23% in clades 1, 2, and 3, respectively) than in the core genes (2.69 ± 0.01%; Supplementary Table 8). Estimations of gene and TE density in the 3-kb sequences flanking each orthologous gene indicated that TE density was significantly greater in the genomic regions surrounding the clade-specific genes than surrounding core genes (*P* < 1 × 10^−10^), even though the density of clade-specific genes was significantly lower than that of core genes (Fig. 3e; *P* < 1 × 10^−10^). Thus, TEs were more widely distributed in the genomic regions that differentiated the three clades. Therefore, these elements might have contributed to clade formation. Indeed, synteny analysis of the genomic regions around the clade-specific orthologous genes in representatives of the three clades (C1.1, C2.1, and C3.1) suggests two non-mutually exclusive ways that TEs might have mediated the formation of clade-specific genes and within-species differentiation (Fig. 3f–g). TEs may have transposed into genes, resulting in insertional mutagenesis and the loss of the affected gene function [41]. Alternatively, TEs insert new genes in the forms of larger transposons that carry cargo genes (for example, the *mer* operon) flanked by transposases. The genomic variants corresponding to the examples shown in Fig. 3f–g were verified in vent and seep mussels by PCR and agarose gel electrophoresis, demonstrating the reliability of the genome assembly (Supplementary Fig. 15).

### Plasmids carry genes encoding important functions for environmental adaptation

**Fig. 4:**
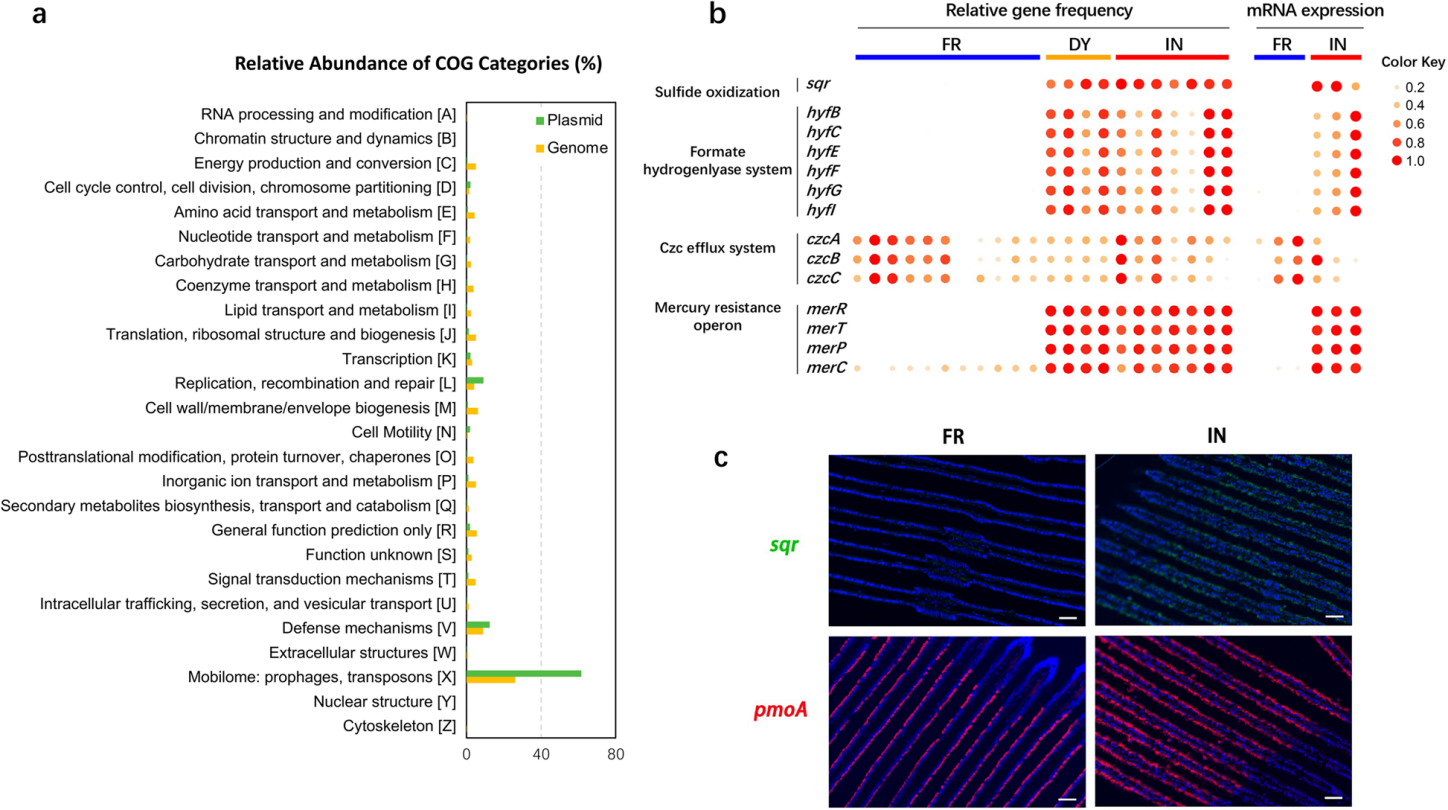
Functional characterization of genes carried by symbiont plasmids. **a** COG categories enriched in plasmid genes and metagenome-assembled genes. **b** Heatmap showing the relative gene frequency and mRNA expression patterns of plasmid genes in individual mussels collected from the hydrothermal vents (Daiyon-Yonaguni Knoll, DY; Iheya North Knoll, IN) and the methane seep (Formosa Ridge, FR). **c** Section showing gill filaments stained using FISH with the gene-specific probes *sqr* (green) and *pmoA* (red), followed by counterstaining with DAPI (blue). The *sqr* probe was ubiquitous in the bacteriocytes of the vent mussel (IN), while the *pmoA* probe was detected in both the vent (IN) and seep (FR) mussels. Scale bar: 50 μm

Plasmids are another important type of MGE that play an important role in bacterial ecology and evolution because they mobilize accessory genes within and between species of bacteria by horizontal gene transfer. We assembled the plasmid sequences from each PacBio HiFi sequencing library using unassembled clean data and predicted 552 contigs carrying 4838 nonredundant genes (Supplementary Table 9). Functional annotation revealed that the plasmid genes were enriched in the COG categories “mobilome: prophages, transposons,” “defense mechanisms,” and “replication, recombination and repair.” Such genes, including plasmid replication initiator protein TrfA and plasmid stabilization system protein ParE, facilitate replication and transfer among microbes (Fig. 4a; Supplementary Table 10). Analysis of gene contents across individual mussels showed that several plasmid genes related to environmental fitness, including sulfide adaptation, heavy-metal resistance, and pH homeostasis, were differentially abundant between the vent- and seep-associated mussels or were environment specific (Fig. 4b). The mRNA expression patterns of these genes were strongly correlated with gene frequency (Fig. 4b), suggesting that differences in gene content underlie the observable differences in gene expression levels.

The sulfide oxidizing gene, sulfide:quinone oxidoreductase (*sqr*), was found only in the vent mussels (Fig. 4b). In our previous metatranscriptomic analysis, we identified the *sqr* gene as differentially expressed between vent and seep mussels; this gene participates in the detoxification of environmental sulfide and improves the fitness of mussel hosts living in sulfide-rich vent habitats [28]. Here, the complete sequence of the *sqr* plasmid was assembled, demonstrating extrachromosomal derivation of the *sqr* gene (Supplementary Fig. 16). FISH analysis with a probe specific to the *sqr* gene provided further evidence that the *sqr* plasmid is abundant in endosymbionts from vent mussels (IN) but not in those from seep mussels (FR; Fig. 4c). Plasmids also contain genes related to heavy-metal resistance, including the *mer* operon for Hg resistance and the Czc (cadmium, zinc, and cobalt) efflux system, which mediates resistance to Co^2+^, Zn^2+^, and Cd^2+^ via cation efflux [42]. The protein sequence encoded by the plasmid *mer* operon was 100% identical to that encoded by the genome assembly *mer* operon, and the *mer* operon was only expressed by the vent-associated symbionts. Similarly, the hydrogenase (*hyf*) operon, which is involved in the maintenance of cellular pH homeostasis, was found only in vent-associated symbionts (Fig. 4b). In contrast, the contents and expression patterns of genes associated with the Czc efflux system were similar between the vent and seep mussel symbionts. Therefore, they may represent a metal-resistance mechanism shared across environments. A detailed description of the plasmid genes can be found in Supplementary Note 7.

## Discussion

It has become increasingly appreciated that intraspecies diversity in symbiotic bacteria has profound consequences for habitat adaptation in deep-sea animals. However, characterizing within-species heterogeneity is challenging for natural populations. In this study, we constructed high-resolution genome assemblies using a refined genome binning pipeline to provide information on the endosymbionts of a deep-sea mussel, *G. platifrons*, which inhabit distinct habitats (hydrothermal vents and methane seeps). We identified clear genetic divergence across the assembled endosymbiotic genomes of *G. platifrons*. The genomes formed three well-differentiated environment-related clades, which were demonstrated to be comparable with those identified from SNPs markers (Supplementary Fig. 13). Although our approach still has limitations, such as resolving highly similar symbionts, we were able to more clearly identify within-species variation compared with traditional binning methods (Supplementary Note 2, Supplementary Table 3).

### The divergence of the vent and seep clades is related to local environmental conditions

The three environment-related endosymbiont clades encode heterogeneous genes related to adaptation to their local geochemical environment. In particular, within the two vent-associated clades, we identified specific genes involved in toxic metal resistance, including genes regulating Hg and Cu homeostasis, which may participate in detoxifying toxic metals and reducing the accumulation of excess ions. Heavy metal concentrations are typically higher at hydrothermal vents than methane seeps due to the interaction between the heated seawater and magmatic rocks [43–45]. In agreement, higher metal concentrations have been measured in the gill and mantle tissues of *G. platifrons* inhabiting hydrothermal habitats in the Okinawa Trough than in those methane seeps in the South China Sea [29] (Fig. 1). Elevated levels of heavy metals can be highly toxic to the host cell. Therefore, the homeostatic regulation and detoxification of heavy metals are likely critical for the survival and proliferation of the vent-associated holobiont.

We also identified clade-specific differences in the utilization of environmental substrates and metabolite biosynthesis related to the geochemical environment. A comparison of nitrate respiration pathways among the three clades suggests that NO_3_^−^ utilization via the DNRA pathway is more efficient in the vent clades than in the seep clade, indicating that the vent-associated symbionts generate additional electrons and ammonium to compensate for lower levels of NO_3_^−^ and higher ambient carbon-to-nitrogen (C/N) ratios. Conversely, the relative inefficiency of the DNRA and denitrification pathways in the seep-associated symbionts may be an adaption to the higher levels of NO_3_^-^ in this environment. Gluconeogenesis-related pathway also differed between environments: the *pckA* gene, critical for gluconeogenesis, was only detected in the seep clade, suggesting that this clade better utilizes and accumulates the carbon and electrons generated from methane in the seep environment.

The functional differences revealed underscore the importance of within-species functional differentiation. Furthermore, comparing pN/pS distributions between the core and clade-specific genes indicates that the latter genes are less conserved, and that functional variations among endosymbiotic bacteria are subject to selection pressure from the local environment. Our results, therefore, highlight the effects of habitat chemistry on adaptive evolution. Additionally, our observations further underscore that within-species variation should be considered when evaluating the impact of deep-sea mining [46].

### The function of TEs in the adaptive evolution of endosymbionts

TEs are important MGEs that are ubiquitous in all organisms. They can move within a single DNA molecule and among different DNA molecules [47] and are a fundamental evolutionary force shaping genome structure and driving genome evolution [15, 18]. TEs are enriched in many horizontally transmitted deep-sea symbionts, including symbiotic bacteria of deep-sea mussels, worms, and snails [22–25]. The proportion of transposases annotated in *G. platifrons* symbionts is relatively lower than that in symbionts of deep-sea snail *Gigantopelta aegis* [25]. TE expansions are more commonly observed in symbionts that recently transitioned to an obligate, host-associated lifestyle, compared to symbionts with a long evolutionary history of association with their hosts [48–51]. Therefore, the difference in TE content might be related to how long ago the symbiotic relationship was established. In addition, the genome assembly of the *G. platifrons* endosymbionts performed in this study also helps clarify the distribution of TEs across the genome and the participation of these MGEs in genome evolution. Specifically, our analysis shows that TEs (as ISs) are more common in clade-specific genes than in core genes. Moreover, the ISs are not stochastically interspersed across the genome but are more abundant in the genomic regions around clade-specific genes (Fig. 3e). These results suggested that TEs might mediate genome evolution by promoting the formation of clade-specific genes.

To clarify the causal relationship between TEs and the within-species functional differentiation of the endosymbiotic bacterium, we analyzed the synteny of representatives from the three clades (C1.1, C2.1, and C3.1). We show that clade-specific gene generation and genome differentiation might arise through TE-mediated gene loss (pseudogenization and other non-functionalization) and TE-mediated functional gene acquisition (Fig. 3f–g). Our analyses suggest that short TEs (ISs), which only contain genes involved in transposition (i.e., a transposase flanked by short, inverted terminal repeat sequences), played a role in gene loss. These TEs can be transposed into gene sequences, inducing insertional mutagenesis and resulting in loss of function (Fig. 3f). Within-species TE-induced pseudogenization, an altered gene repertoire has also been observed in genomes of the aphid symbiont *Hamiltonella defensa* [52]. Multiple TE insertions may even induce partial or complete losses of orthologous genes in particular clades (Fig. 3f). Similar examples of TE expansion and pseudogenization have been reported in many recently evolved symbionts and are considered indicative of gene loss and genome reduction [49, 53, 54]. Moreover, more complex TEs (i.e., transposons) often carry additional cargo genes that convey functions that improve survival in certain environments, such as antibiotic- and metal-resistance genes [16]. For example, previous studies have shown that transposons are transferred horizontally among bacteria, leading to the expression of new genes and functions in the infected bacteria [47]. We propose that TEs of *G. platifrons* symbionts also allowed new functions by altering the cargo gene repertoire (Fig. 3g). Here, the *mer* operon specific to clade 1 was shown to have been inserted as a transposon. This is consistent with studies showing that the *mer* operon is often localized on transposons, and that these TEs have disseminated mercury resistance across bacteria from a wide range of environments [55, 56]. Thus, TE expansion has helped to increase genomic plasticity and functional heterogeneity in the *G. platifrons* endosymbiont through gene disruptions, deletions, and insertions. Genomic plasticity and heterogeneity have, in turn, facilitated efficient intraspecies differentiation in response to environmental selection pressure.

### Plasmids are also important for symbiont adaptive evolution

Genetic heterogeneity in bacteria is not limited to the differentiation of the chromosomal genomes but is also manifested as genetic differences among plasmids. Plasmids are extrachromosomal MGEs that are widely distributed across bacteria. Plasmids may carry genes conferring important functions, such as resistance to antibiotics or heavy metals, on the bacterial host, improving host fitness under the corresponding adverse ecological conditions [57, 58]. Unlike chromosomal DNA, which is limited to vertical transmission from mother cells to daughter cells, plasmids can be transmitted horizontally among bacterial species. Therefore, plasmids represent potential access to a vast reservoir of bacterial community genes and are an important source of evolutionary innovation in bacteria [57, 59, 60], including symbiotic bacteria [52, 61]. For instance, plasmids in the bacterial endosymbionts of aphids (plant sap-sucking insects) encode genes essential for the biosynthesis of amino acids and vitamins and prevent nutrition deficiencies in the aphid host [20, 62].

Despite the known importance of plasmids in bacterial evolution, the roles of plasmid genes in deep-sea symbioses remain unexplored. Here, we constructed the first assembly of plasmid sequences from deep-sea endosymbionts from different habitats. Several plasmid genes related to environmental adaptation were differentially expressed between habitats, including genes involved in sulfide oxidization, heavy metal resistance, and pH homeostasis. Thus, our results highlight the importance of plasmids for the adaption of symbiotic partners to highly sulfidic, heavy-metal rich, and acidic deep-sea environments and suggest that plasmids play an indispensable role in the acquisition of genetic innovation and the stimulation of adaptative evolution in deep-sea horizontally transmitted endosymbionts.

### Mosaic adaptive evolution of bacterial endosymbionts in deep-sea mussels

**Fig. 5:**
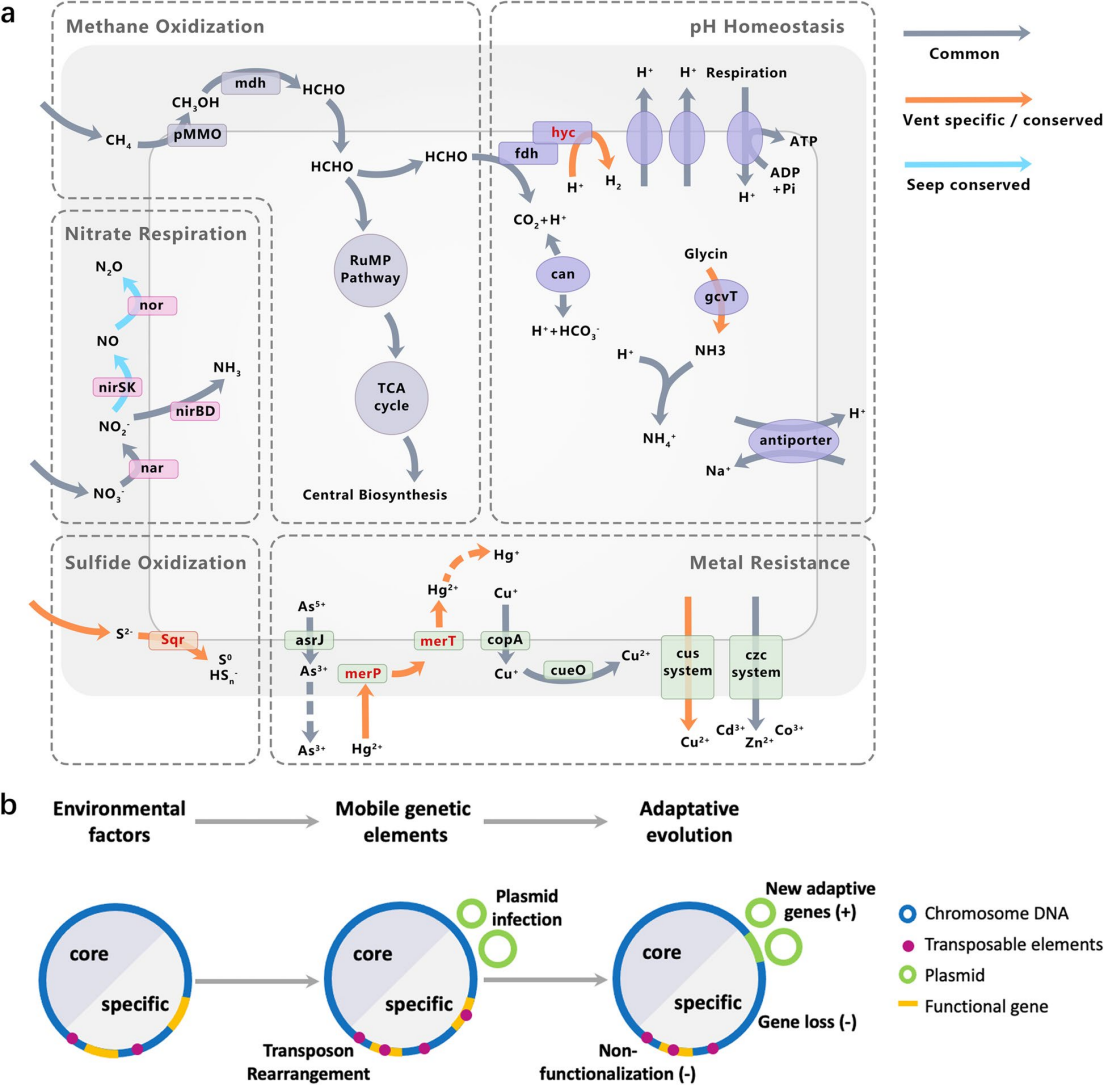
Genetic heterogeneity and environment-driven adaptative evolution in the symbiotic bacteria of *G. platifrons*. **a** Schematic showing the core and clade-specific metabolic pathways involved in environmental adaptation, as indicated by metagenomic comparison. Gray arrows indicate pathways common to the genomes of both vent and seep mussel symbionts. Blue arrows indicate pathways only conserved across the seep mussel symbionts, while orange arrows indicate pathways only conserved across or detected in the vent mussel symbionts. Genes only expressed in the vent symbionts are shown in red text. **b** Model of the predicted mosaic pattern of adaptive environmental evolution in the symbionts of *G. platifrons*. The environment is the main extrinsic force maintaining genome plasticity and driving within-species differentiation, while MGEs are an intrinsic source of genetic innovation via TE rearrangement and plasmid infection. The MGE-mediated loss and gain of functional genes facilitate the adaptative evolution of symbiont functional genomes

Marked within-species variation of its endosymbiotic bacteria, including variable gene content and genome architecture, was detected in *G. platifrons*. Consistent with this, a previous study showed that horizontally transmitted symbionts in marine environments are characterized by large genomes, substantial functional variation, and high recombination rates, while vertically transmitted symbionts had low recombination rates and degraded genomes [2]. Notably, genetic variation was not evenly distributed across genome regions or genes. The selection pressure on core genes was significantly lower than on clade-specific genes, suggesting that the core genes undergo purifying selection while clade-specific genes undergo accelerated evolution. This result reflects the essential conservation of certain core functions across the methane-oxidizing endosymbionts, including methane oxidization, the biosynthesis of amino acids and cofactors, and secretion systems. These functions enable nutrient synthesis and the transportation of nutrients from the bacterium to the host and are critical for effective symbiosis [27, 40]. Conversely, genes encoding adaptive-related functions are “hot spots” of evolutionary change. High heterogeneity is maintained, and genetic innovations are introduced in these genes through TE expansion of chromosomal genomes and horizontal gene transfer via plasmids, facilitating the rapid acquisition of beneficial adaptive genes in response to environmental selection pressure (Fig. 5). Therefore, the endosymbionts of *G. platifrons* exhibit a mosaic evolutionary pattern: core genes essential for the central metabolism and symbiosis maintenance are conserved. In contrast, clade-specific genes confer environmental solutions and evolve rapidly due to MGEs.

## Conclusions

In this study, we performed a fine-scale metagenomic analysis to characterize within-species compositional and functional heterogeneity of the bacterial symbionts of *G. platifrons* inhabiting the markedly different hydrothermal vent and methane seep habitats. The genomes of the symbiotic bacteria were highly heterogeneous and have undergone frequent genome rearrangement. Our phylogenetic analysis suggests that they fall into three distinct, habitat-linked clades. They are mosaic—comprising core genes essential for symbiosis interspersed with adaptive genes. The core genes were conserved across the clades, while the expression patterns and functions of the adaptive genes were habitat specific. MGEs play a critical role in the formation and functional differentiation of adaptive genes via TE insertions, some caused partial or complete loss of orthologous genes, and some introduced new functional genes. In addition to the habitat-specific functional differences among chromosomal genes, our results demonstrate the importance of extrachromosomal plasmids in acquiring adaptive innovations. Functional heterogeneity of adaptive genes benefits the symbionts by utilizing diverse energy substrates and increases their fitness by improving resistance and tolerance to potential environmental stressors. We also propose a model for the mosaic adaptative evolution of the conserved core genes and heterogeneous adaptive genes of horizontally transmitted endosymbionts in response to changing environmental conditions. In this model, environmental factors act as the main extrinsic force driving within-species variation, while MGEs maintain genome plasticity and introduce genetic innovations through frequent rearrangements and horizontal gene transfer.

## Methods

### Sample collection and measurement of environmental geochemistry

The *G. platifrons* specimens used for metagenomic sequencing were obtained from a methane seep in the South China Sea (FR: 22°06′N, 119°17′E) and two hydrothermal vents in the middle and southern portions of the Okinawa Trough (IN: 27°47′N, 126°53′E and DY: 24°51′N, 122°42′E). Immediately after being taken on board, the symbiont-containing gill tissues of the sampled mussels were removed and stored at −80 °C for DNA extraction. For fluorescent *in situ* hybridization (FISH), the freshly collected gill filaments were fixed in cold 4% paraformaldehyde at 4 °C overnight. After washing 3 times in phosphate buffer saline (PBS), the fixed gill filaments were dehydrated in 75% ethanol and stored at −20 °C until use.

The environmental parameters of site FR and site IN were measured *in situ* at the time of collection. Environmental parameters at site DY were not measured due to the limitations of that cruise. At each site, the *in situ* temperature of the seawater was measured using a Seabird SBE 25 plus conductivity-temperature-depth sensor (CTD) (SeaBird Electronics, Inc., USA). The *in situ* methane and the dissolved oxygen (DO) concentrations were obtained using a CH_4_ sensor (CONTROS HydroC CH_4_ sensor, Kongsberg Gruppen, Norway), and a Seabird SBE 43 dissolved oxygen sensor (Sea-Bird Electronics, Inc., USA), respectively. Seawater samples around the chemosynthetic ecosystems were obtained with a multi-sampler manipulated by the ROV *Faxian*. Fluids were collected in 150-mL gas-tight samplers to recover both liquid and gaseous samples. The fluid samples were processed as quickly as possible after the ROV returned onboard. The pH of the seawater was measured directly using a pH meter (Thermo Scientific Orion 5-star, USA) and an electrode (Ray Magnetic E-201-D, China). Hydrogen sulfide (H_2_S) concentrations in the collected fluids were measured using the methylene blue method. Nitrate (NO_3_^−^) concentrations were determined colorimetrically using a QuAAtro continuous flow analyzer (SEAL Analytical Ltd., UK); nitrate was reduced to nitrite using a Cu-Cd reduction coil and was then detected as a red complex.

### DNA extraction and metagenome sequencing

Symbiotic bacterial DNA was extracted from the mussel gills following the method described by Li et al. (2020) [63]. Briefly, the gill tissues were homogenized with PBS buffer, and the tissue debris in the supernatant were removed by sequential filtration through 10-μm, 5-μm, and 3-μm Millipore nitrocellulose membranes. Next, the bacterial cells were collected by centrifugation at 8000 rpm for 5 min. Bacterial genomic DNA was extracted from the bacterial pellets using an EZNA D3350 bacterial DNA kit (Omega Bio-tek, Norcross, GA, USA).

Sequencing reactions were performed on both the Illumina platform and the PacBio system (Supplementary Table 1, Supplementary Fig. 1). In total, 22 individuals from three sites were sequenced using the Illumina X10 platform: 11 from FR, 7 from IN, and 4 from DY. PacBio sequencing was performed on additional five samples (2 from FR and 3 from IN) using circular consensus sequencing (CCS) mode. Individuals from DY were not used for PacBio sequencing due to the limited number of organisms available. The CCS reads were generated using the CCS protocol of SMRTLink version 6.0 with default settings. Only CCS reads fitting HiFi quality standards (3+ full-length passes and average read quality scores > Q20) were retained. Summaries of the reads generated for each sample can be found in Supplementary Table 1.

### Construction of metagenome-assembled genomes (MAGs) using Illumina data

Low-quality reads were trimmed and filtered using Fastp [64]. The taxonomic composition of the microbiome associated with the host was recovered using PhyloFlash with default parameters [65]. The quality-controlled reads were assembled using metaSPAdes v3.1 with kmer sizes of 21, 33, 55, 71, 91, and 101 [66]. Long contigs (> 1000 bp) were clustered into bins with a modified MetaWRAP pipeline [67] that refined the binning results from MaxBin2 [68], MetaBat2 [69], CONCOCT [70], VAMB [71], SolidBin [72], and BinSanity [73]. The taxonomic classification of each MAG was determined using the Genome Taxonomy Database Toolkit (GTDB-Tk) [74]. To determine the relative abundance of each MAG, we used an in-house Python script to calculate the number of reads mapped per Kb of MAG divided by Gb of the corresponding metagenome (RPKG).

### High-resolution endosymbiotic genome assembly using a refined binning pipeline

Before assembly, we first filtered the PacBio reads to retain only those derived from endosymbionts. A database of the genomes of the host (GenBank accession no. JAOEFJ000000000, unpublished data) and the gill-associated microbiomes (MAGs assembled using Illumina data) was used as a reference for the filter. All high-quality PacBio reads were mapped against this database using Magic-BLAST [75] with default parameters (minimum identity, 98%; coverage, 80%). The reads mapped to reference sequences belonging to *G. platifrons* and to microorganisms other than the genus *Methyloprofundus* were excluded from further analysis.

Genome construction was performed separately for each PacBio dataset. An overview of the workflow for assembly is shown in Supplementary Fig. 1. Because the high intraspecific heterogeneity and abundant TEs hampered the assembly and binning of the microbe genomes at the strain level, we develop a refined binning pipeline to cluster the PacBio reads from different strains to facilitate high-resolution genome assembly. Briefly, for each PacBio dataset, we realigned the Illumina clean reads and calculated the mapping depth of all high-quality PacBio long reads. Due to the high genome sequence similarity of strains, a strict criterion (mismatch < 3) for mapping was used, and only primary hits (either unique- or multi-match sequences) with the highest mapping scores were kept for the calculation of mapping depth. Based on the assumption that reads from the same strain should have the same sequencing depth, we clustered the PacBio long reads into subdatasets by sequencing depth for subsequent assembly. In addition to sequencing depth, we also considered similarities in GC content and kmer composition. This method of clustering reads into subdatasets, each theoretically containing reads of a single strain, was implemented in CONCOCT [70] based on our pilot studies. Further preliminary analyses showed that Flye-meta [76] outperformed Canu [77] and FALCON [78] with respect to contig N50 and genome completeness. Thus, the clustered subdatasets were separately assembled into genomes using Flye-meta [76] with the options for metagenome selected. The assembled genomes were binned using the modified MetaWRAP pipeline [67] to remove possible contamination if necessary. The quality of each MAG was evaluated using CheckM [79].

The refined genome construction pipeline was validated using four test datasets with simulated PacBio and Illumina reads from published *E. coli* strains (Supplementary Note 2; Supplementary Tables 2 and 3). For the simulation of the CCS dataset, PBSIM3 [80] was used through multi-pass sequencing of the generated CLR reads with a pass number of 8 (https://github.com/yukiteruono/pbsim3). The HiFi reads were generated using ccs software with default parameters. Simulated pair-end Illumina data was generated using wgsim [81] with a base error rate of 0.01, a mutation rate of 0.1, and an insert size of 150 bp. The binning and assembly of the simulated datasets were implemented as described above.

### Functional annotation of the genome assemblies

The final MAGs were predicted and annotated with the NCBI Prokaryotic Genome Annotation Pipeline (PGAP). All genes were also searched against the NCBI nr database, the Kyoto Encyclopedia of Genes and Genomes (KEGG), and the Clusters of Orthologous Genes (COG) database. Gene Ontology (GO) functional mappings were assigned based on the nr annotations using OmicBox. COG categories were assigned based on the definitions on the COG website (https://www.ncbi.nlm.nih.gov/research/COG) using an in-house python script.

### Phylogenetic reconstruction and ANI analysis

The pangenome of the endosymbionts was constructed using OrthoFinder [82] with stringent blastp parameters (0.98 identity and 0.8 coverage for both query and subject sequences). Orphan genes and representative coding sequences (the longest sequences) for the orthologs were combined to generate the pangenome using an in-house pipeline. Orthologous proteins with more than two copies were aligned with PRANK and trimmed with TrimAl [83]. Separate gene trees were constructed based on the final dataset using maximum likelihood (ML) and the CAT + GTR substitution model in RAxML [84]. Local posterior probabilities (LPP) were utilized to gauge the confidence of each branch. Finally, a species tree was estimated from the best-scoring ML trees for each gene using ASTRAL-MP [85]. To assess inter-genome similarity, we calculated pairwise ANI values among genome assemblies with the dRep pipeline [86]. Samples were clustered and visualized based on average ANI values using a heatmap drawn with the pheatmap R package.

### Estimation of evolutionary rate

The branch-free (b_free) model of the ETE 3 package [87] was used to estimate the evolutionary rate along each lineage of the three clades. Because outlier genes with larger dN/dS may generate deviations in evaluating the overall selective constraint in species, our dataset was filtered to remove genes with dN/dS > 4 as in the previous study [88]. The lineage-specific dN/dS values were calculated for each ortholog and for 1000 concatenated alignments constructed from 25 randomly selected orthologs, respectively. Genes with outlier values (dN/dS < = 0.0001 or > 4) were filtered from the resulting codeml outputs [88, 89]. The Wilcoxon rank-sum test was applied to calculate the significance levels of the evolutionary rates of each lineage.

### Identification and functional enrichment analysis of core- and clade-specific orthologs

Core genes were defined as orthologous genes shared in more than 90% of all the genomes. Cluster (clade)-specific genes were defined as those orthologs uniquely present and overrepresented in one clade. We initially identified orthologous genes whose frequency in one cluster (clade) was significantly greater than that of the other genomes (Fisher’s test, adjusted *P* < 0.05), and then intersected genes were uniquely present in that clade. Gene Ontology (GO) analysis and Kyoto Encyclopedia of Genes and Genomes (KEGG) pathway enrichment analysis were carried out using the clusterProfiler R package [90].

### Population genetics analysis

To validate the genome assemblies and to characterize differences in endosymbiont populations among individual mussels, we performed genome-wide SNP analyses of the methylotrophic symbionts with the pangenome as the reference. SNPs were called as described in Ansorge et al. (2019) [8], using adjusted scripts (https://github.com/rbcan/MARsym_paper) compatible with the updated GATK4 pipeline [91] and Python 3. In brief, quality-controlled short reads were mapped to the reference pangenome with a minimum identity of 97%. The reads surrounding the indels were realigned in GATK4. SNPs were called using GATK HaplotypeCaller with ploidy set to 10, and unreliable SNPs were removed using GATK VariantFiltration with the following settings: *QD* < 2, *FS* > 60, *MQ* < 40, MQRankSum < −20, and ReadPosRankSum < −8. The fixation index *F*_ST_ was calculated for each gene using a modified script from the *Bathymodiolus brooksi* symbiont repository (https://github.com/deropi/BathyBrooksiSymbionts/tree/master/Population_structure_analyses). Mean pairwise FST values were plotted on a heatmap, and individuals were clustered accordingly using the pheatmap R package. We also performed PCoAs of the reconstructed ploidy composition of each SNPs to visualize the relationships among individuals from the three sites.

### Assessment of pN/pS

To explore selection pressures on the orthologs, values of pN/pS, a variant of dN/dS that can be used based on intraspecies SNVs, were estimated. The dN/dS values use the number of SNVs in an alignment, while the pN/pS values use SNV frequencies in their calculations. Estimation of pN/pS values were following the methods of Romero Picazo et al. [13], with updated scripts to fit python3 and GATK4 from the *Bathymodiolus brooksi* symbiont repository (https://github.com/deropi/BathyBrooksiSymbionts). The calculated number of synonymous and nonsynonymous variants was normalized by the potential number of synonymous and nonsynonymous variants. Besides, we added 1 to the number of observed synonymous mutations in each gene to circumvent the limitation of undefined estimates of genes with no synonymous mutations, which is a standard correction for dN/dS ratios [92]. Finally, we compared pN/pS values between cluster-specific and core genes to determine whether positive selection pressure on the cluster-specific genes was greater. Plots were constructed, and nonparametric *t*-tests were calculated in R.

### IS distribution among different genome regions

ISs were predicted using ISfinder (https://www-is.biotoul.fr/index.php) [93]. To test whether the insertion of transposes tends to be less common in the regions surrounding core genes, which are more conservative in functions, we calculated the frequency of IS occurrence in the 3-kb regions flanking the core and clade-specific genes. The coordinates of the window boundary were parsed from the GFF annotations using pybedtools [94]. The frequency tests were statistically analyzed using SciPy [95].

### Plasmid mining

To recover the plasmid dataset as intact as possible, we reassembled the high-quality long reads with only host-originated reads, which were obtained using Flye [76] with the plasmid and metagenome options selected. The coding genes were predicted using Prokka [96] with default parameters. The coding sequences obtained were searched against the *Methyloprofundus* datasets using blastn (requiring 0.95 identity and 0.8 coverage for both query and subject sequences). Contigs containing more than three *Methyloprofundus* genes and having a relative proportion of *Methyloprofundus* genes greater than 30% were regarded as candidate endosymbiont sequences. Phage sequences were predicted by analyzing these contigs using Phigaro [97], and plasmid sequences were predicted using PlasFlow [98]. Contigs were categorized as plasmid sequences only if the sequences were positively predicted by PlasFlow and negatively predicted by Phigaro. The obtained plasmid sequences were further filtered by removing relatively short (< 5000 bp) and low-coverage (< 1/3 of genome coverage) sequences.

### Assessment of gene content variation

To identify functional differences in endosymbiont populations between seep- and vent-associated mussels, we developed a gene-based method to calculate gene content in each of the mussel groups. Briefly, all the assembled genes, including the genes in the assembled genomes as well as genes in plasmids, were clustered using CD-HIT-EST [99], requiring protein sequences with 0.98 consistency to remove sequence redundancy and construct a nonredundant gene set. The Illumina clean reads were aligned against the gene set using Magic-BLAST [75], with a percent identity cutoff of 98%. Gene frequencies in the individual mussels were calculated using the featureCounts program and normalized with sequencing depth and gene length.

### Metatranscriptomic analysis

The metatranscriptomic data were obtained previously [28]. The quality-controlled clean metatranscriptomic data were aligned against the nonredundant gene set using Magic- BLAST [75] with a 98% identity cutoff. The featureCounts program [100] was used to generate a count matrix of RNA sequences from the metatranscriptomic. The expression levels of all identified genes were then quantified with the fragments per kilobase per million mapped fragments (FPKM) metric using the FPKM_count.py function in the RSeQC R package [101].

### Fluorescence in situ hybridization (FISH)

Gill sections were dehydrated and embedded in Paraplast Plus (Sigma-Aldrich) following standard protocols. Sections (7 μm thick) were cut using a microtome (Leica). Fragments of *pmoA* and *sqr* were amplified using standard PCR reactions with gene-specific primer pairs (pmoA-Fw: 5′-AACTGGTGGTGACTGGGATTT3′, pmoA-Rv: 5′-TAGATGCCTTCGCCACTAATG3′; sqr-Fw: 5′-TCATCCATTCAAGTGTGACCTG-3′, sqr-Rv: 5′-AATCGGCTATTGGTTCTGGG-3′) and *G. platifrons* gill DNA as template. The *pmoA* and *sqr* amplicon lengths were 621 bp and 937 bp, respectively. The PCR fragments were ligated into T-Vector pMD 20 plasmids (Takara) and Sanger sequenced to confirm insertion. After purification, the cloning vectors were used as templates for digoxigenin-labeled probe synthesis using a PCR DIG Probe Synthesis Kit (Roche).

FISH experiments were performed following Ikuta et al. [7]. In brief, sections were de-waxed in xylene and rehydrated in a decreasing ethanol series. The rehydrated sections were washed in PBS twice for 10 min each time. For permeabilization, sections were incubated with 10 μg/ml proteinase K at 37 °C for 10 min, and the digestion was stopped by washing twice in PBST for 5 min each time. Next, the sections were post-fixed with 4% paraformaldehyde in PBS for 20 min, followed by washing twice in PBS for 5 min each time. To denature the DNA, the sections were incubated in 70% formamide in 2× SSC at 72 °C for 3 min, after which the samples were dehydrated (in 70%, 95%, and 100% ethanol at −20 °C) and air-dried. The digoxigenin-labeled gene probe was simultaneously denatured at 80 °C for 15 min in a hybridization mix (50% formamide, 2× SSC, 10% dextran sulfate, 0.2 μg/μl sheared salmon sperm DNA, 0.15% SDS) at a final concentration of 5 ng/μl. The denatured probes were chilled on ice until application to the air-dried sections. The hybridization reaction was carried out at 37 °C for at least 16 h in a moist chamber. After the hybridization step, the unbonded probes were removed by washing twice in 20% formamide and 0.1× SSC at 37 °C for 30 min each time, twice in 2× SSC at 37 °C for 15 min each time, and twice in PBST for 15 min each time. The sections were then incubated in 0.5% blocking reagent in PBST for 30 min and overnight in a 1/1000 volume of anti-digoxigenin POD (Roche) in PBST containing 0.5% blocking reagent at 4 °C. Next, the redundant reaction reagents were washed three times in PBST for 10 min each time and twice in TNT buffer (100 mM Tris-HCl pH 7.5, 150 mM NaCl, 0.05% Tween 20) for 5 min each time. To amplify the hybridization signals, 1/50 cyanine-3 (Perkin Elmer) in 1× Plus Amplification Diluent (Perkin Elmer) mix was applied to the *pmoA-*hybridized sections and fluorescein tyramide (Perkin Elmer) in 1× Plus Amplification Diluent (Perkin Elmer) as applied to the *sqr*-hybridized sections. The sections were then incubated at room temperature for 30 min in a moist chamber. After washing three times in TNT and three times in PBST, the sections were mounted using ProLong Diamond Antifade Reagent with DAPI (Thermo Fisher Scientific).

### Supplementary Information

**Additional file 1:** **Supplementary Note 1.** Composition of functional bacterial communities in gills of *Gigantidas platifrons*. **Supplementary Note 2. **Validation of the refined metagenome binning approach using test datasets of *Escherichia coli* strains. **Supplementary Note 3.** Assessment of the genome recovery rate of the refined genome assembly pipeline. **Supplementary Note 4.** Selection analysis among the genomes from the three clades. **Supplementary Note 5.** Population differentiation between endosymbiotic strains in different mussel groups. **Supplementary Note 6.** Detailed description of the functional difference between the vent and seep endosymbiont clades involved in environmental adaptation. **Supplementary Note 7.** Detailed description of the plasmid genes related to environmental adaptation. **Supplementary Fig. 1.** Overview of the refined binning pipeline in this study for the strain-level genome assembly using both Illumina and PacBio sequencing data. **Supplementary Fig. 2.** Taxonomic analysis of Illumina-produced sequences from 22 individual mussels. **Supplementary Fig. 3.** ML phylogenetic tree (left) and the pairwise ANI values (right) of the reference *E. coli *genomes and the assembled bins from test datasets 1 and 2 (strains with low ANI) using different binning pipelines. **Supplementary Fig. 4.** ML phylogenetic tree (left) and the pairwise ANI values (right) of the reference *E. coli *genomes and the assembled bins from test datasets 3 to 4 (strains with high ANI) using different binning pipelines. **Supplementary Fig. 5.** Synteny of the assembled bins obtained from *E. coli* test datasets 1 to 4 with their best hit reference genomes. **Supplementary Fig. 6.** The reads recovery rate of PacBio (a) and Illumina (b) sequencing datasets. **Supplementary Fig. 7.** ML phylogenetic reconstruction of endosymbiont strains based on orthologous genes conserved across the pangenome. **Supplementary Fig. 8.** Heatmap of pairwise average nucleotide identities (ANI) of genome assemblies in the three clades. **Supplementary Fig. 9.** Whole genome alignments of representative endosymbiotic genomes in the three clades. **Supplementary Figure 10.** Box plot of the dN/dS values for each clade obtained from each ortholog. **Supplementary Figure 11.** Heatmap showing the pairwise fixation index (*F*_ST_) value among individual mussels collected from the hydrothermal vents (Daiyon-Yonaguni Knoll, DY; Iheya North Knoll, IN) and the methane seep (Formosa Ridge, FR). **Supplementary Fig. 12.** Principal coordinate analysis (PCoA) of individual mussels from the hydrothermal vents (Daiyon-Yonaguni Knoll, DY; Iheya North Knoll, IN) and the methane seep (Formosa Ridge, FR). **Supplementary Fig. 13.** Phylogenetic relationship of symbiont strains reconstructed with DESMAN (a) and their relative abundance among individuals from the vent (Daiyon-Yonaguni Knoll, DY; Iheya North Knoll, IN) and seep (Formosa Ridge, FR) sites (b). **Supplementary Fig. 14.** Box plot showing dN/dS values in the core genes and the clade-specific genes of the three clades. **Supplementary Fig. 15.** PCR amplification using gill DNA demonstrating the genomic variants among genomes from different clades. **Supplementary Fig. 16.** The assembled plasmid encoding sulfide:quinone oxidoreductase (*sqr*) gene.

**Additional file 2:** **Supplementary Table 1.** Information of Gigantidas platifrons samples used in metagenomic analysis and summary of sequencing data. **Supplementary Table 2.** Test datasets created from *E. coli* strain genomes. **Supplementary Table 3.** Summary of the assembled bins obtained by different binning methods using the* E. coli* test datasets. **Supplementary Table 4.** Summary of the genome features of the assembled strains. **Supplementary Table 5.** Functional enrichment analysis of core orthologs present more than 90 % strains**Supplementary Table 6.** Functional enrichment analysis of clade specific orthologs. **Supplementary Table 7** Number of environmental-related orthologs in each genome and their percentage in each clade. **Supplementary Table 8.** Summary of transposase in the core genes and clade-specific genes of the assembled genomes. **Supplementary Table 9.** Summary of plasmids assembled from PacBio sequencing libraries. **Supplementary Table 10.** COG and KEGG functional annotation of the non-redundant plasmid genes.

## Data Availability

The PacBio and Illumina sequencing data have been deposited in the NCBI’s SRA database under BioProject no. PRJNA891367 and PRJNA891060, respectively. All scripts for the bioinformatics analyses are available in GitHub (https://github.com/CODRbio/StrainSybionts_mussel).
