# Supplementary material for: Mosaic environment-driven evolution of the deep-sea mussel Gigantidas platifrons bacterial endosymbiont
Source: Microbiome. 2023 Nov 16;11:253. doi: 10.1186/s40168-023-01695-8 (PMC10652631; doi:10.1186/s40168-023-01695-8)
Supplement: Supplementary file 2 — Additional file 1: Supplementary Note 1. Composition of functional bacterial communities in gills of Gigantidas platifrons. Supplementary Note 2. Validation of the refined metagenome binning approach using test datasets of Escherichia coli strains. Supplementary Note 3. Assessment of the genome recovery rate of the refined genome assembly pipeline. Supplementary Note 4. Selection analysis among the genomes from the three clades. Supplementary Note 5. Population differentiation between endosymbiotic strains in different mussel groups. Supplementary Note 6. Detailed description of the functional difference between the vent and seep endosymbiont clades involved in environmental adaptation. Supplementary Note 7. Detailed description of the plasmid genes related to environmental adaptation. Supplementary Fig. 1. Overview of the refined binning pipeline in this study for the strain-level genome assembly using both Illumina and PacBio sequencing data. Supplementary Fig. 2. Taxonomic analysis of Illumina-produced sequences from 22 individual mussels. Supplementary Fig. 3. ML phylogenetic tree (left) and the pairwise ANI values (right) of the reference E. coli genomes and the assembled bins from test datasets 1 and 2 (strains with low ANI) using different binning pipelines. Supplementary Fig. 4. ML phylogenetic tree (left) and the pairwise ANI values (right) of the reference E. coli genomes and the assembled bins from test datasets 3 to 4 (strains with high ANI) using different binning pipelines. Supplementary Fig. 5. Synteny of the assembled bins obtained from E. coli test datasets 1 to 4 with their best hit reference genomes. Supplementary Fig. 6. The reads recovery rate of PacBio (a) and Illumina (b) sequencing datasets. Supplementary Fig. 7. ML phylogenetic reconstruction of endosymbiont strains based on orthologous genes conserved across the pangenome. Supplementary Fig. 8. Heatmap of pairwise average nucleotide identities (ANI) of genome assemblies in the three clades. [file 40168_2023_1695_MOESM1_ESM.docx]

***Supplementary Information for:***

**Mosaic environment-driven evolution of the deep-sea mussel *Gigantidas platifrons* bacterial endosymbiont**

Yan Sun^1,2,3#^, Minxiao Wang^1,2,3#^, Lei Cao^1,2,3^, Inge Seim^4,5^, Li Zhou^1,2,3^, Jianwei Chen^6^, Hao Wang^1,2,3^, Zhaoshan Zhong^1,2,3^, Hao Chen^1,2,3^, Lulu Fu^1,2,3^, Mengna Li^1,2,3^, Chaolun Li^1,2,3,7,8^*, Song Sun^1,2,3,8^*

^1^ CAS Key Laboratory of Marine Ecology and Environmental Sciences, and Center of Deep Sea Research, Institute of Oceanology, Chinese Academy of Sciences, Qingdao 266071, China

^2^ Laboratory for Marine Ecology and Environmental Science, Qingdao National Laboratory for Marine Science and Technology, Qingdao 266071, China

^3^ Center for Ocean Mega-Science, Chinese Academy of Sciences, Qingdao 266071, China

^4^ Integrative Biology Laboratory, College of Life Sciences, Nanjing Normal University, Nanjing 210046, China

^5^ School of Biology and Environmental Science, Queensland University of Technology, Brisbane QLD 4000, Australia

^6^ BGI Research-Qingdao, BGI, Qingdao 266555, China

^7^ South China Sea Institute of Oceanology, Chinese Academy of Sciences, Guangzhou 510301, China

^8^ University of Chinese Academy of Sciences, Beijing 10049, China

^#^These authors contributed equally to the study

*Corresponding authors:

Chaolun Li, lcl@qdio.ac.cn; Song Sun, sunsong@qdio.ac.cn

**This PDF file includes:**

**Supplementary Notes 1-7**

**Supplementary Figures 1-16**

**Supplementary References**

**Other suppl. materials include an Excel file:**

**Supplementary Tables 1-10**

**Contents**

[Supplementary Notes 4](#_Toc142381693)

[Supplementary Note 1: Composition of functional bacterial communities in gills of *Gigantidas platifrons* 4](#_Toc142381694)

[Supplementary Note 2: Validation of the refined metagenome binning approach using test datasets of *Escherichia coli* strains 4](#_Toc142381695)

[Supplementary Note 3: Assessment of the genome recovery rate of the refined genome assembly pipeline. 6](#_Toc142381696)

[Supplementary Note 4: Selection analysis among the genomes from the three clades. 6](#_Toc142381697)

[Supplementary Note 5: Population differentiation between endosymbiotic strains in different mussel groups 7](#_Toc142381698)

[Supplementary Note 6: Detailed description of the functional difference between the vent and seep endosymbiont clades involved in environmental adaptation 8](#_Toc142381699)

[Supplementary Note 7: Detailed description of the plasmid genes related to environmental adaptation 9](#_Toc142381700)

[Supplementary Figures 11](#_Toc142381701)

[Supplementary Figure 1: Overview of the refined binning pipeline in this study for the strain-level genome assembly using both Illumina and PacBio sequencing data. 11](#_Toc142381702)

[Supplementary Figure 2: Taxonomic analysis of Illumina-produced sequences from 22 individual mussels. 12](#_Toc142381703)

[Supplementary Figure 3: ML phylogenetic tree (left) and the pairwise ANI values (right) of the reference *E. coli* genomes and the assembled bins from test datasets 1 and 2 (strains with low ANI) using different binning pipelines. 13](#_Toc142381704)

[Supplementary Figure 4: ML phylogenetic tree (left) and the pairwise ANI values (right) of the reference *E. coli* genomes and the assembled bins from test datasets 3 to 4 (strains with high ANI) using different binning pipelines. 14](#_Toc142381705)

[Supplementary Figure 5: Synteny of the assembled bins obtained from *E. coli* test datasets 1 to 4 with their best hit reference genomes. 18](#_Toc142381706)

[Supplementary Figure 6: The reads recovery rate of PacBio (a) and Illumina (b) sequencing datasets. 19](#_Toc142381707)

[Supplementary Figure 7: ML phylogenetic reconstruction of endosymbiont strains based on orthologous genes conserved across the pangenome. 20](#_Toc142381708)

[Supplementary Figure 8: Heatmap of pairwise average nucleotide identities (ANI) of genome assemblies in the three clades. 21](#_Toc142381709)

[Supplementary Figure 9: Whole genome alignments of representative endosymbiotic genomes in the three clades. 22](#_Toc142381710)

[Supplementary Figure 10: Box plot of the dN/dS values for each clade obtained from each ortholog. 22](#_Toc142381711)

[Supplementary Figure 11: Heatmap showing the pairwise fixation index (*F*_ST_) value among individual mussels collected from the hydrothermal vents (Daiyon-Yonaguni Knoll, DY; Iheya North Knoll, IN) and the methane seep (Formosa Ridge, FR). 23](#_Toc142381712)

[Supplementary Figure 12: Principal coordinate analysis (PCoA) of individual mussels from the hydrothermal vents (Daiyon-Yonaguni Knoll, DY; Iheya North Knoll, IN) and the methane seep (Formosa Ridge, FR). 23](#_Toc142381713)

[Supplementary Figure 13: Phylogenetic relationship of symbiont strains reconstructed with DESMAN (a) and their relative abundance among individuals from the vent (Daiyon-Yonaguni Knoll, DY; Iheya North Knoll, IN) and seep (Formosa Ridge, FR) sites (b). 24](#_Toc142381714)

[Supplementary Figure 14: Box plot showing dN/dS values in the core genes and the clade-specific genes of the three clades. 25](#_Toc142381715)

[Supplementary Figure 15: PCR amplification using gill DNA demonstrating the genomic variants among genomes from different clades. 26](#_Toc142381716)

[Supplementary Figure 16: The assembled plasmid encoding sulfide:quinone oxidoreductase (*sqr*) gene. 27](#_Toc142381717)

[Supplementary References 28](#_Toc142381718)

# Supplementary Notes

## Supplementary Note 1: Composition of functional bacterial communities in gills of *Gigantidas platifrons*

Twenty-two mussel individuals, including 11 from Formosa Ridge (FR), 7 from Iheya North Knoll (IN), and 4 from Daiyon-Yonaguni Knoll (DY), were sequenced using the Illumina platform (Supplementary Table 1) to overview the composition of gill-associated bacteria and their abundance in different sampling sites. Taxonomical analysis based on 16S rRNA read frequencies recovered by PhyloFlash [1], as well as the frequencies of metagenomic reads that mapped to the metagenome assembled genomes (MAGs), revealed similar bacteria composition in both vent and seep mussels (Supplementary Fig. 2). Assembly of Illumina metagenomic sequencing data obtained all the draft genomes of main bacterial taxa indicated by 16S rRNA analysis (Supplementary Fig. 2). As indicated in the results, a single phylotype of methanotrophic belong to *methyloprofundus*, the genus represented as the uncultivated methane-oxidizing bacterial endosymbionts of deep-sea bathymodiolin mussels [2], made up the vast majority of the microbial community (96.10 - 98.17 % detected by average MAG coverage, supplementary Fig. 2b). The second most abundant taxon was Helicobacteraceae belonging to Campylobacterota, which had been described as common episymbiotic sulfur-oxidizing bacteria in bathymodiolin mussels [3, 4]. Besides, 16S rRNA coverage revealed similar results to our previous study that the abundance of Campylobacterota in vent mussels collected in site IN was more than that of site FR, possibly contributing to the adaption of the sulfide-rich environment in that site [5]. Intriguingly, we assembled three Campylobacterota genomes, one of which was enriched in mussel samples from IN site. These results suggested possible genetic or functional diversity of Campylobacterota bacteria under the selection of different environmental factors.

## Supplementary Note 2: Validation of the refined metagenome binning approach using test datasets of *Escherichia coli* strains

Two mocked PacBio datasets were generated to evaluate the performance of the reads-binning-based MAG reconstruction pipeline in unveiling the within-specific divergences (Supplementary Table 2). Each PacBio dataset consists of four subsets (corresponding to PacBio data 1-4 in Supplementary Table 2). One is comprised of all six strains with even abundance, while the other three subsets were generated based on the estimated strain compositions from DESMAN [6] to simulate the real situation in this study (Supplementary Note 5; Supplementary Fig. 13). To test the performance of the pipeline, the two mocked PacBio datasets were stimulated with two scenarios using different *E. coli* strains. In the first dataset, the reads were generated from six *E. coli* strains with relatively lower ANI values, ranging from 96.33 to 98.85, representing a simpler population to distinguish (Supplementary Table 2; Supplementary Fig. 3). The other dataset was simulated from six strains with relatively higher ANI values, ranging from 98.73 to 99.61, to test the performance for the relatively complex situation (Supplementary Table 2; Supplementary Fig. 4). For each PacBio dataset, there are also two Illumina datasets to provide the coverage information for reads binning. One corresponds to an ideal situation that includes six Illumina samples (Illumina data 11-16), each containing only one strain, while the other represents a more realistic scenario without these one-strain-only Illumina data. Overall, our tests were set to compare the assembly efficiency for datasets with different complexity of strain composition and coverage information. The four test datasets were numbered Test Datasets 1 to 4. Among them, Test Datasets 1 and 2 were simulated from strains with relatively lower ANI values; Test Datasets 3 and 4 were from higher similar strains. Test Datasets 1 and 3 included the additional six simulated Illumina data containing reads from only one strain (Illumina data 11-16). Detailed setups of the four test datasets are listed in Supplementary Table 2.

For each test dataset, the four PacBio datasets were assembled separately following the refined pipeline in Supplementary Fig. 1. Briefly, the PacBio reads were clustered into subdatasets (clusters of reads from the same strain) for subsequent assembly based on similarities in GC content, kmer composition, and Illumina reads’ mapping depth. Besides, we also use the conventional binning approach by refined MetaWRAP [7] with additional binners, including MetaBat2 [8], CONCOCT [9], MaxBin2 [10], and SolidBin [11], for a comparison of the results.

The retrieved bins of the four test datasets using different binning approaches were summarized in Supplementary Table 3 and Supplementary Fig. 3-4. For Test Datasets 1 and 2 composed of strains with relatively lower ANI values, the performance of our refined binning pipeline and the conventional binning method were comparable (Supplementary Fig. 3; Supplementary Table 3). Both binning approaches assembled all the strains from the corresponding sequencing libraries. The genomes obtained by the refined method had lower contamination levels compared to the genomes obtained by conventional methods. However, the genomes obtained by conventional methods had higher completeness and N50 values. For test datasets 3 and 4, which were composed of more similar strains with higher ANI values, the genome recovery rate of our refined binning pipeline was significantly improved than the conventional binning method (Supplementary Fig. 4; Supplementary Table 3). Using the refined binning pipeline, we successfully assembled genomes of all the strains in Test Dataset 3 and 85 % of all the strains in Test Dataset 4, except for the two highly similar strains with ANI values greater than 99.6 (ATCC8739 and BL21). Although the resolving power for such highly similar strains was slightly inferior, the refined assembly approach outperformed the conventional binning approach, which only recovered 50 % and 15 % of all the strains in Test Dataset 3 and 4, respectively.

In terms of the quality of the assembly, our pipeline successfully clarified the reads from different strains for subsequent assembly, as demonstrated by the proportions of correctly clustered reads (Supplementary Table 3). In both the test datasets composed of strains with relatively lower and higher ANI values, an average of 99.60% and 93.38% of the reads in the subdatasets, respectively, were derived from their corresponding strains (Supplementary Table 3). This indicates that the pipeline effectively separated the reads from different strains, ensuring accurate assembly results. Besides, synteny analysis was conducted between the assembled bins and their reference genomes. The result revealed a high structure and sequence similarity between assembled bins and the reference genomes in all four test datasets (Supplementary Fig. 5), demonstrating the confidence of the assembled sequences.

The results of the test datasets demonstrate that our metagenome binning pipeline is effective in obtaining relatively complete strain composition and high-quality strain-level genomic information, especially for datasets composed of highly similar strains, which outperforms conventional binning methods in terms of genomic assembly (Supplementary Fig. 4; Supplementary Table 3). Despite having certain limitations, such as relatively lower completeness and N50 values in the assembly of less similar strains, the refined approach provides a valuable supplement to the traditional methods and is helpful in obtaining more genomic information on highly similar strains for subsequent functional analysis.

## Supplementary Note 3: Assessment of the genome recovery rate of the refined genome assembly pipeline.

We use both the PacBio and the Illumina data to assess the genome recovery rate of our refined genome assembly pipeline. First, we constructed *G. platifrons* methanotrophic endosymbionts genome database (MOXdb) by combining all the published genomes from the NCBI public database and integrating the genome assemblies generated using PacBio and Illumina data available herein. Subsequently, all reference genomes generated by our new custom pipeline from each sample or location were combined as a test dataset to check whether our protocol can recover the actual biodiversity of the methanotrophic symbionts. The PacBio long-reads and Illumina short-reads obtained in this study were aligned to the candidate reference genome using minimap2 [12], with secondary alignments allowed. During the alignment, the low-quality mapping was filtered using a Python script RPKG_fromBam_minimap2.py (available in GitHub, see “Availability of data and materials”), which selected reads with a global identity above 98% and coverage above 90% for Illumina short reads and 75% for HiFi long reads. The reads recovery rate was then calculated by dividing the number of reads aligned to the PacBio-assembled genomes (test dataset) by those aligned to the reference genome (MOXdb). The results are shown in Supplementary Fig. 6. An average of 67.7% of the PacBio reads can be utilized in the genome assembly pipeline, and an average of 71.6% of the Illumina reads can be mapped to PacBio-assembled genomes. The results provided insights into the effectiveness of the PacBio-assembled genomes and their representation in capturing the complete genomic diversity of *G. platifrons* methanotrophic endosymbionts. It is worth noting that the coverage threshold for PacBio alignment was set high in this analysis, which may have led to lower utilization rates of the dataset. Furthermore, since the MOXdb used in this study included web resources without extensive quality control, there is a possibility of host contamination being introduced.

## Supplementary Note 4: Selection analysis among the genomes from the three clades.

Selection analysis was conducted to identify positively selected genes among the three clades. The results revealed 239, 110, and 159 orthologous genes under positive selection in strains from clade 1, clade 2, and clade 3, respectively. Many of these genes encode important functions involved in metabolite biosynthesis and environmental adaptation. In particular, orthologous genes positively selected in clade 1 included genes participating in metal resistance (arsenate reductase, *arsC*, and Cu^+^ translocating P-type ATPase, *copA*) and cellular pH homeostasis (cation:H^+^ antiporter). Besides, we also detected heat-inducible transcriptional repressor HrcA (*hrcA*) was under positive selection in clade 1. HrvA controls the transcription of GroE and DnaK chaperones, therefore, works as a regulatory gene in the defense mechanism against a sudden heat-shock stress [13]. In the seep-type strains in clade 3, genes participating in fatty acid biosynthesis were positively selected, including beta-ketoacyl-acyl carrier protein synthase I (*fabB*), the chain elongation condensing enzyme that controls the fatty acid composition and influences the rate of fatty acid production [14]. Additionally, coproporphyrinogen III oxidase (*hemF*), a gene involves in biosynthesis of cofactors, was also a positively selected gene in clade 3. The positive selection of fatty acid and cofactors biosynthesis-related genes may be involved in the abundant carbon and electrons stably generated from methane in the seep environment.

## Supplementary Note 5: Population differentiation between endosymbiotic strains in different mussel groups

To validate the genomes assembled using the refined binning pipeline and to evaluate genetic differences in endosymbiont populations among individual mussels. We performed genome-wide SNP analyses of the methylotrophic symbionts as described in previous studies [15, 16] and calculated the pairwise fixation index (*F*_ST_) value among individual mussels based on SNPs in the orthologous genes. The results revealed a habitat-associated population differentiation of the samples, with a lower *F*_ST_ value within individuals of the same habitat, in contrast with a higher *F*_ST_ value among individuals from different habitat types (Supplementary Fig. 11). Notably, *F*_ST_ value was much higher among the vent mussels (0.117-0.709) than that among the seep mussels (0.088-0.277). This result could be explained by the co-occurrence of strains from two different clades, which showed highly variated abundance among the vent mussel individuals (as results indicated in Fig. 2e in the main text), highlighting the environmental heterogeneity in the vents. For the vent mussels, individuals having a similar distribution of symbiont abundance were having lower *F*_ST_ values than those dominated by different clades. Individuals dominated by strains of clade 2 (DY1, DY2, and IN4) and clade 1 (DY3, IN1, and IN5) had the highest pairwise *F*_ST_ values (0.577 to 0.709). Principal coordinate analysis (PCoA) revealed similar results on population differentiation and show clear separation of the symbionts at the vent and seep sites (Supplementary Fig. 12).

In addition, strains were reconstructed based on the SNPs using DESMAN [6]. A total of 13 strains were reconstructed. Most of them (12/13) were phylogenetically clustered into three lineages (Supplementary Fig. 13a), which showed the same phylogenetic relationship with the assembled genomes in our study (Fig. 2a). The distributions of the reconstructed strains across individual mussels were also estimated (Supplementary Fig. 13b). The results showed a distinct difference in strain composition between the vent and seep mussel individuals. The seep mussels were mainly composed of strains from one of the lineages, while the vent mussels were composed of strains from the other two. Notably, the two vent lineages were corresponding to the vent-type clades assembled in our study (clade 1 and clade 2), as their relative abundance in vent individuals shared a similar pattern with that detected based on metagenome assemblies. Among the vent individuals, IN1, IN5, and DY3 were dominated by strains from vent cluster 1 (corresponding to vent-type clade 1), and IN4, DY1, and DY2 were dominated by strains from vent cluster 2 (corresponding to vent-type clade 2). Overall, the results validated the confidence of the genomes assembly of the three-environment-linked clades in our current study and demonstrated the intraspecies differentiation between the endosymbionts in vent and seep mussels.

## Supplementary Note 6: Detailed description of the functional difference between the vent and seep endosymbiont clades involved in environmental adaptation

The functional comparison of genomes from the vent and seep clades revealed marked differences with unique genes involved in adaptation to their local geochemical environments. First, in our current analysis, genomes of the two vent clades contained specific genes involved in toxic metal resistance, including mercury (Hg) and copper (Cu) homeostatic regulatory and resistant genes, which may participate in the detoxification of toxic metal and reduce the accumulation of excess ions. The mercury (Hg) resistance (*mer*) operon was found as clade-specific genes in clade 1. It was composed of the Hg^2+^ responsive activator MerR and transporters MerTPC, which function in active uptake and transport Hg^2+^ into the bacteria cytosol. It is noteworthy that the makeup of *mer* operon in *G. platifrons* was different from the conventional *mer* system, for the lack of mercuric reductase MerA, reducing the toxic, soluble, and bioavailable Hg^2+^ to insoluble metallic mercury (Hg^0^) [17, 18]. However, the conservation of *mer* operon in clade 1 may suggest a novel Hg resistance mechanism in the endosymbiotic bacteria. Cu serves as an important biological co-factor for maintaining cellular processes, but high concentrations of copper are extremely toxic to the cell [19]. The copper homeostatic regulatory network in the endosymbiotic genomes is made up of *cue* system (including Cu^+^ translocating P-type ATPase CopA and multicopper oxidase CueO) and *cus* system (the copper efflux pump CusCBA). The former detoxifies copper through oxidizing cuprous Cu^+^ to cupric Cu^2+^ under low to moderate copper levels and aerobic conditions with the inner membrane CopA and the periplasmic CueO, while the latter provides copper tolerance under extreme copper stress and anaerobic conditions by a copper efflux pump encoded by *cusCBA* operon [20]. Both the *cue* and *cus* systems commonly existed in all three clades, but gene copy numbers were different among them. An additional *cusCBA* operon was expanded in clade 1. Clade 2 also had an extra *cusCBA* operon, but incomplete and partially lost in some of the genomes . Furthermore, three copies of *copA* genes were detected in clades 2 and 3, while only one was in clade 1. The presence of copper regulatory genes in different genomes corresponded to their living environments with those from clades 1 and 2 dominating in vents with a higher concentration of copper.

Differences in utilization of the environmental substrates and biosynthesis among the three clades were also related to their geochemical environment. Gene contents in nitrate respiration pathways of different clades indicate the efficient NO_3_^-^ utilization by the dissimilatory nitrate/nitrite reduction to ammonium (DNRA) pathway in vent clades, while more flexible usages of both DNRA and denitrification pathways were found in seep clade. The biological utilization of the two pathways may be relative to the surrounding carbon-to-nitrogen (C/N) ratios. DNRA is favored under higher ambient C/N ratios while denitrification is upregulated under lower C/N ratios [21]. The concentration of nitrate in hydrothermal vents is lower than that in cold seep, thus the vent strains would be more likely to use an efficient DNRA pathway to get more electrons. Simultaneously, the ammonium generated by DNRA could also be used for biosynthesis, which is a more efficient use of nitrogen sources than the production of NO or N_2_O gas through denitrification. For clade 3 living in the seep environment, reservation of both DNRA and denitrification pathways may be an adaption to the relatively higher levels of NO_3_^-^. Furthermore, *pckA*, the key gene using the TCA cycle intermediate cycle to drive gluconeogenesis, was only conserved in the seep clades. As compared with the vent environment, which contains more electron donors such as sulfide or hydrogen, the seep fluids provide a more stable and unitary electron supply from methane, thus, a conservative gluconeogenesis pathway might be more beneficial for the utilization and accumulation of carbon source in the methane-oxidizing symbionts.

Genes related to the regulation of pH homeostasis also differed between the seep and vent clades, possibly reflecting an increased tolerance of the more acidic vent environment. Genes related to the regulation of pH homeostasis in endosymbiotic strains of *G. platifrons* included Na^+^/H^+^ antiporter (*nhaG*) and glycine cleavage system T-protein (*gcvT*). The Na^+^/H^+^ antiporter NhaG, which drives the electroneutral exchange of H^+^ against Na^+^ ions to ensure pH homeostasis [22, 23], is commonly present in all the strains. GcvT catalyzes the oxidative cleavage of glycine and produces NH_4_^+^ participating in cellular pH homeostasis [24, 25]. Only genomes in the vent clades encode GcvT among the three clades.

## Supplementary Note 7: Detailed description of the plasmid genes related to environmental adaptation

In this study, we assembled the plasmid sequences of the endosymbionts in *G. platifrons* and revealed that the plasmids carry genes encoding important functions for environmental adaptation, including sulfide oxidization, heavy metal resistance, and pH homeostasis, differentially distributed between samples from different environments. Firstly, sulfide:quinone oxidoreductase (*sqr*) gene, which was found involved in the detoxification of sulfide in our previous study in metatranscriptome analysis [5], was identified as a plasmid gene, and the result was evidenced by PCR and FISH experiments. Plasmids also contain genes related to metal resistance, including the *mer* operon and Czc (cadmium, zinc, and cobalt) efflux system.

The *mer* operon, which had been identified in the genome assembly sequences of strains in clade 1, was also detected in the plasmid genes. Synteny analysis of the plasmid and chromosomal *mer* operon revealed that the gene sequences and the organizations of *mer* operon were identical, but their flanking regions were different. The discrepant synteny of *mer* flanking regions proposed the possibility of the reservation of *mer* operon in both chromosomal and extrachromosomal genetic materials in the same strain. Alternatively, the *mer* plasmid may provide a complementary function for the chromosomal genome of other vent-type strains without a *mer* operon and spread across different strains through horizontal gene transfer and TE-mediated transposition. The *mer* operon was specifically expressed in the vent-associated symbionts, revealing its important function in the adaptation to high environmental mercury concentration in vent habitats. In contrast, the contents and expression patterns of genes associated with the Czc efflux system, which mediates resistance to Co^2+^, Zn^2+^, and Cd^2+^ via cation efflux, were similar between the vent and seep mussel symbionts and may therefore represent a metal-resistance mechanism that is shared across environments. Additionally, the hydrogenase (*hyf*) operon was also found in plasmid specifically distributed in vent mussels. The *hyf* operon encoded hydrogenase that interacted with formate dehydrogenase to produce an active formate hydrogenlyase (FHL) complex, which catalyzed the oxidation of formate to carbon dioxide (CO_2_) and hydrogen (H_2_) to help maintain pH homeostasis within the cell [26]. Furthermore, the FHL complex is also identified as energy‑converting hydrogenases linking the H_2_ metabolism and energy conservation under anaerobic conditions [27]. It is coupled with H_2_ metabolism to establish a transmembrane electrochemical ion gradient, which then is utilized by an ATP synthase for energy conservation by generating ATP. Thus, the *hyf* operon may improve the ability to maintain pH homeostasis and generate ATP under anaerobic conditions in vent mussels.

# Supplementary Figures


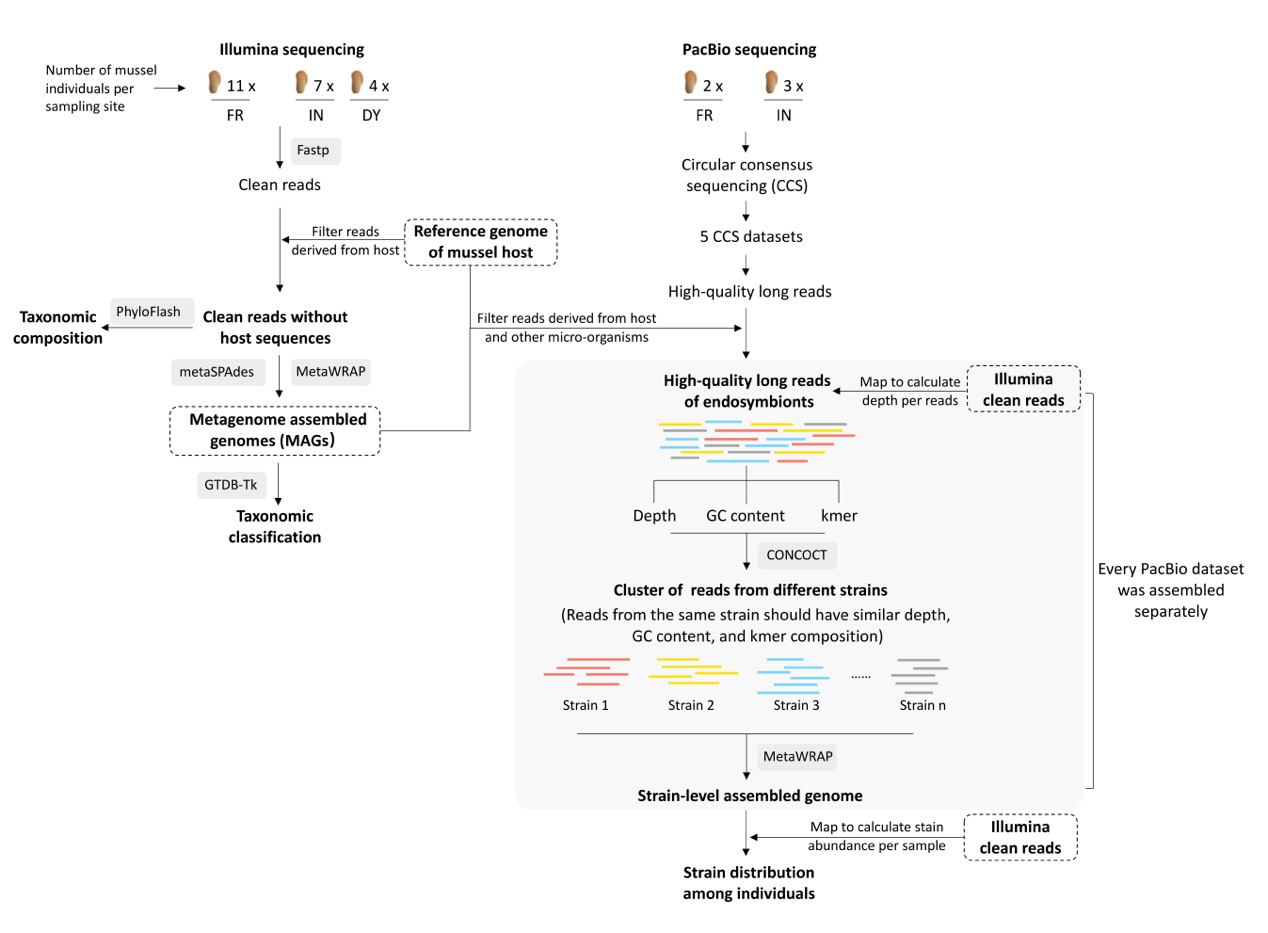


## Supplementary Figure 1: Overview of the refined binning pipeline in this study for the strain-level genome assembly using both Illumina and PacBio sequencing data.


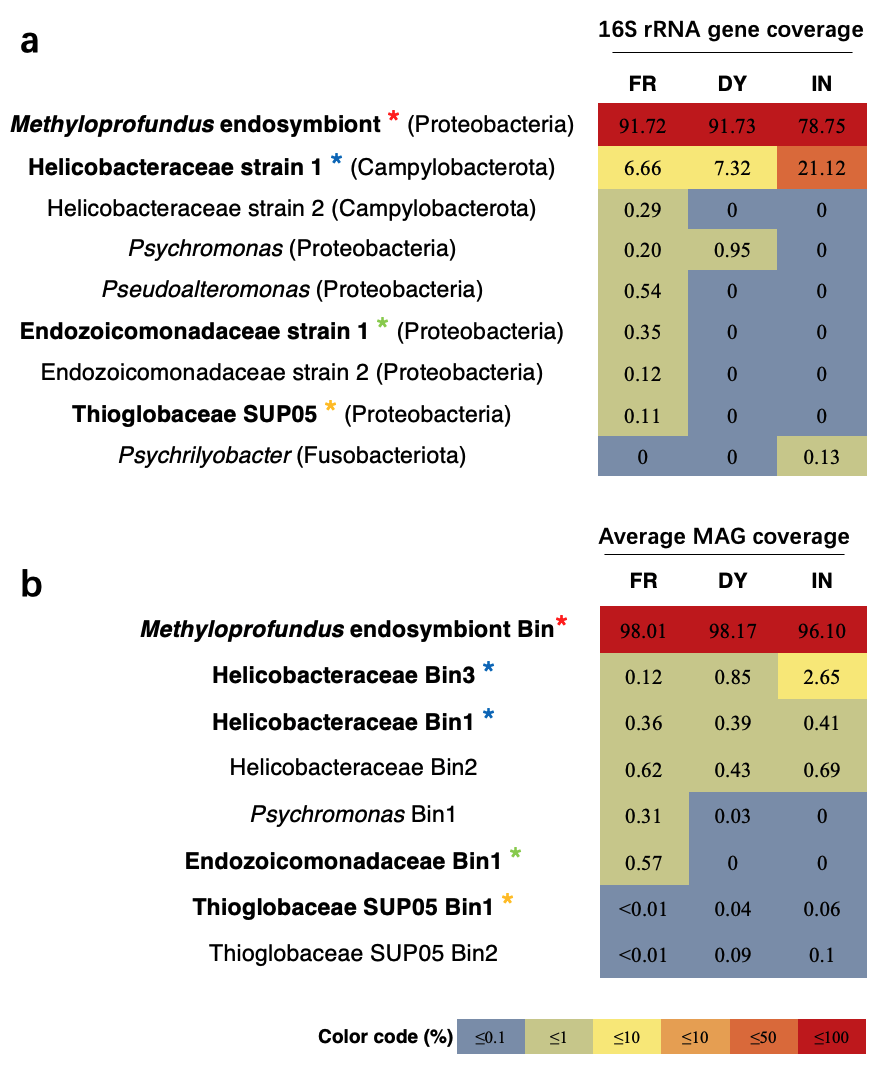


## Supplementary Figure 2: Taxonomic analysis of Illumina-produced sequences from 22 individual mussels.

The results of 16S rRNA read frequencies in the metagenomes (a) and the frequencies of metagenomic reads mapped to the metagenome assembled genomes (MAGs; b) revealed that a single phylotype methane-oxidizing symbionts from the genus of *methyloprofundus* made up the vast majority of bacterial community in both the vent and seep mussels. FS, DY, and IN represent the sampling sites of the methane seep in the South China Sea (Formosa Ridge, FR) and the two hydrothermal vents in the Okinawa Trough (middle: Iheya North Knoll, IN; southern: Daiyon-Yonaguni Knoll, DY), respectively. “*” indicates that the 16S rRNA sequencing could be found in the corresponding MAGs.


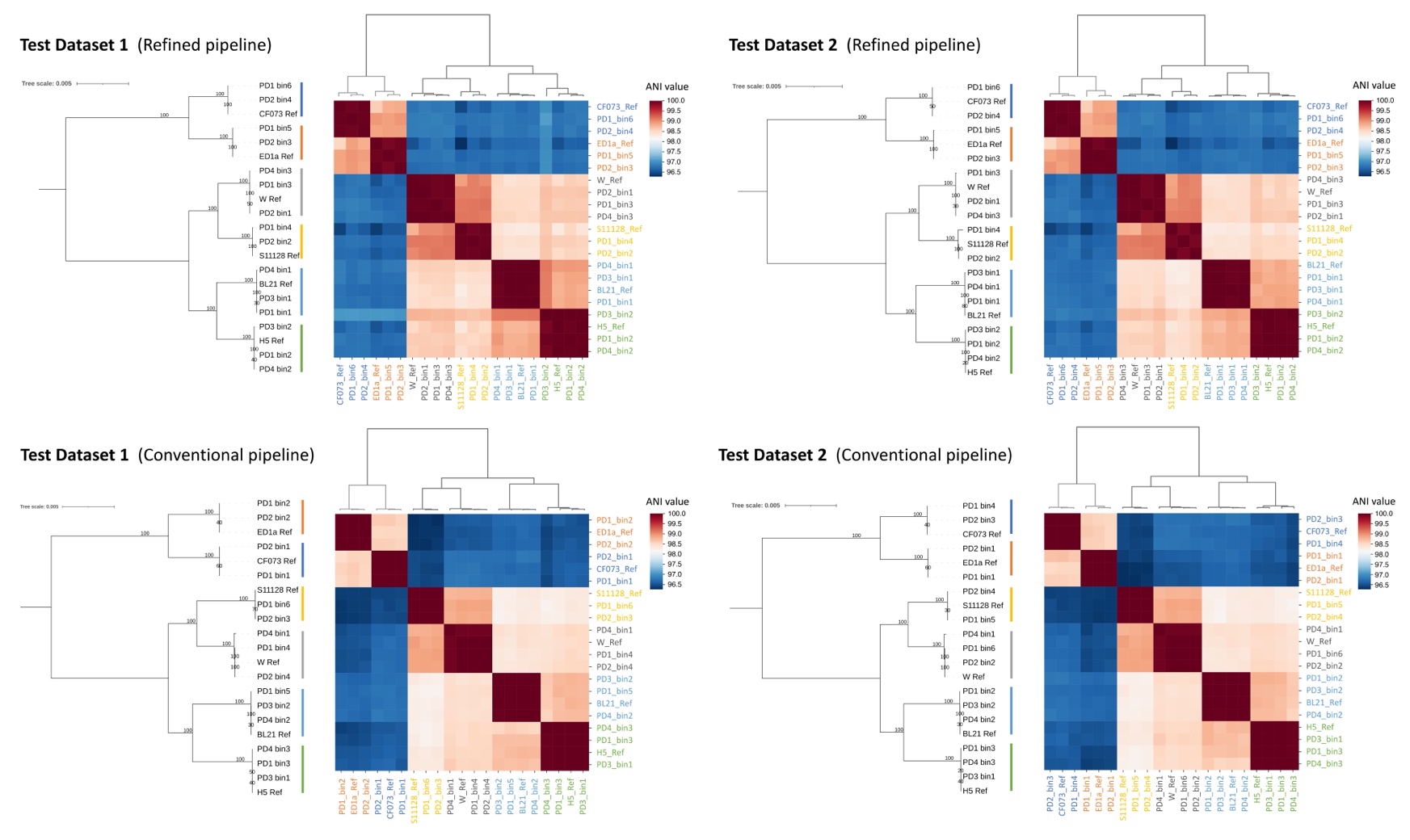


## Supplementary Figure 3: ML phylogenetic tree (left) and the pairwise ANI values (right) of the reference *E. coli* genomes and the assembled bins from test datasets 1 and 2 (strains with low ANI) using different binning pipelines.


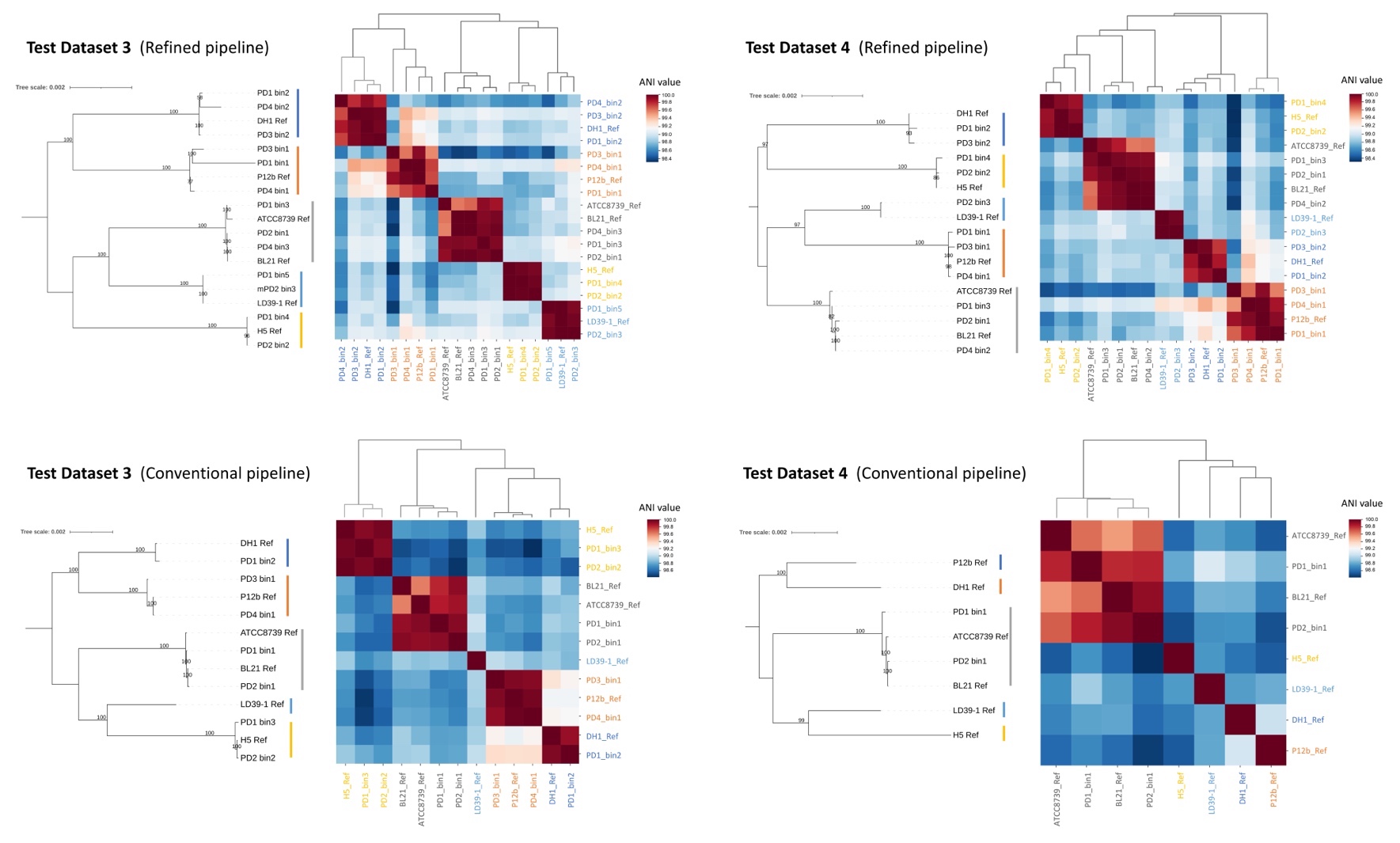


## Supplementary Figure 4: ML phylogenetic tree (left) and the pairwise ANI values (right) of the reference *E. coli* genomes and the assembled bins from test datasets 3 to 4 (strains with high ANI) using different binning pipelines.


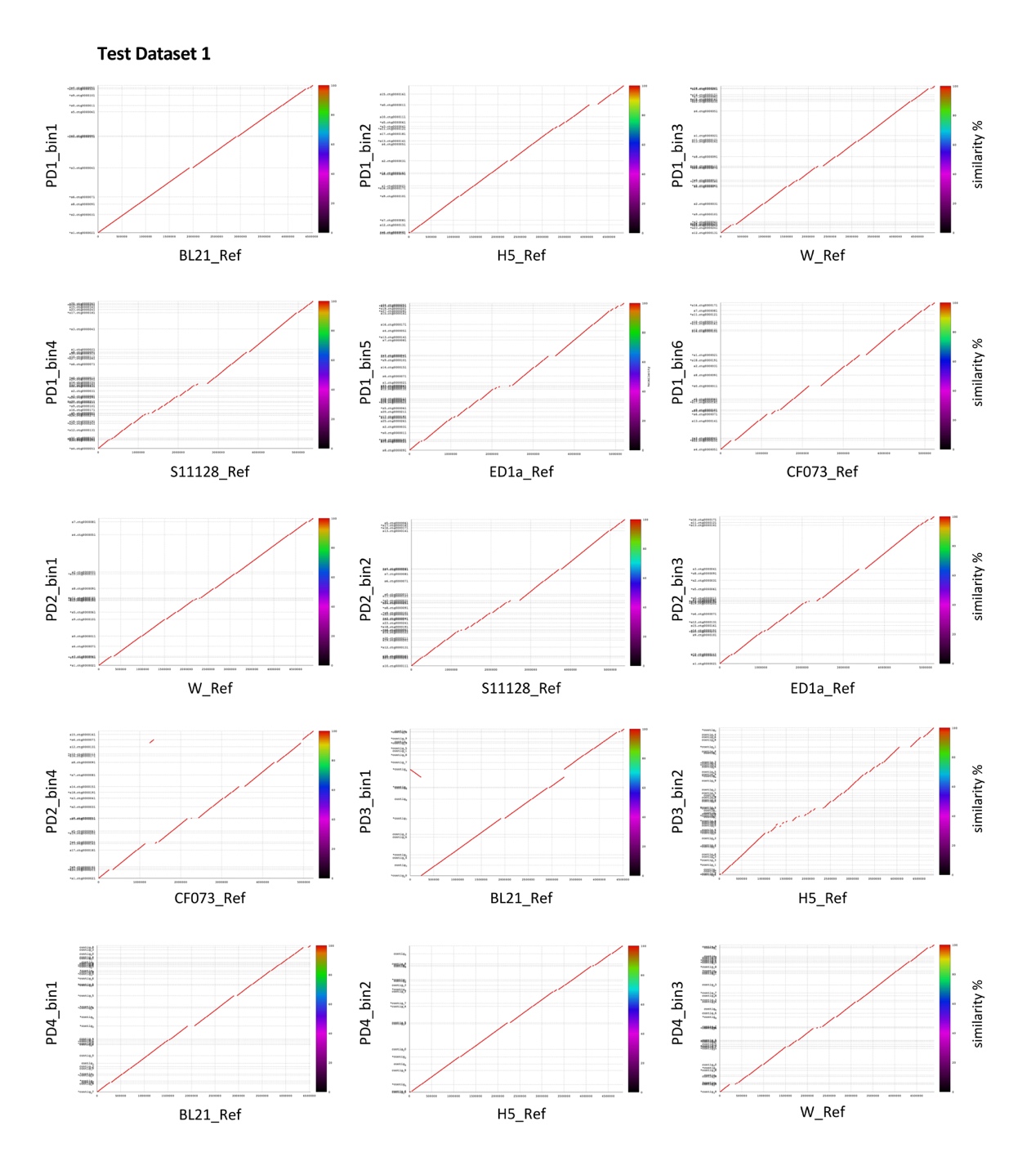


(Continued on the next page)


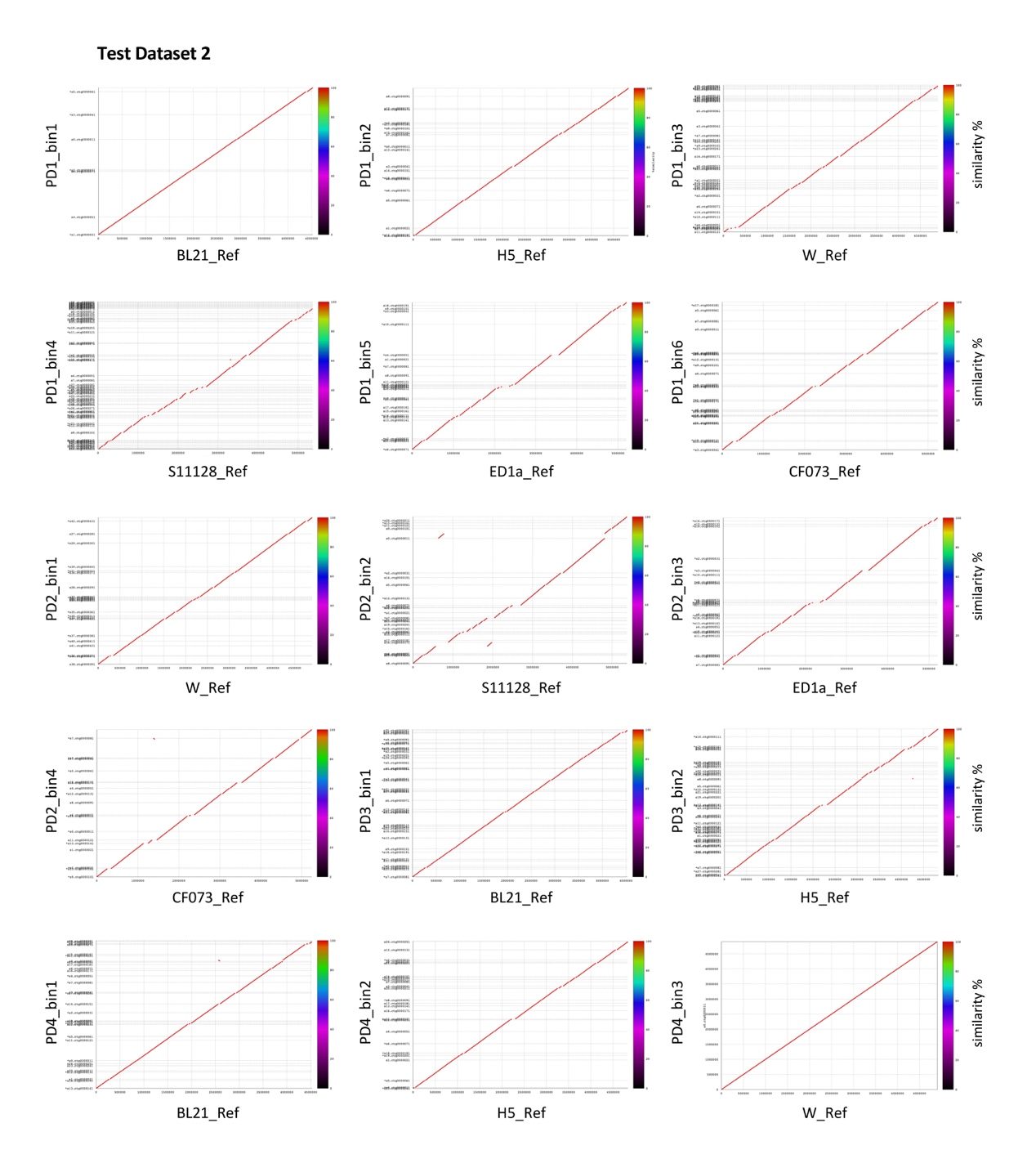


(Continued on the next page)


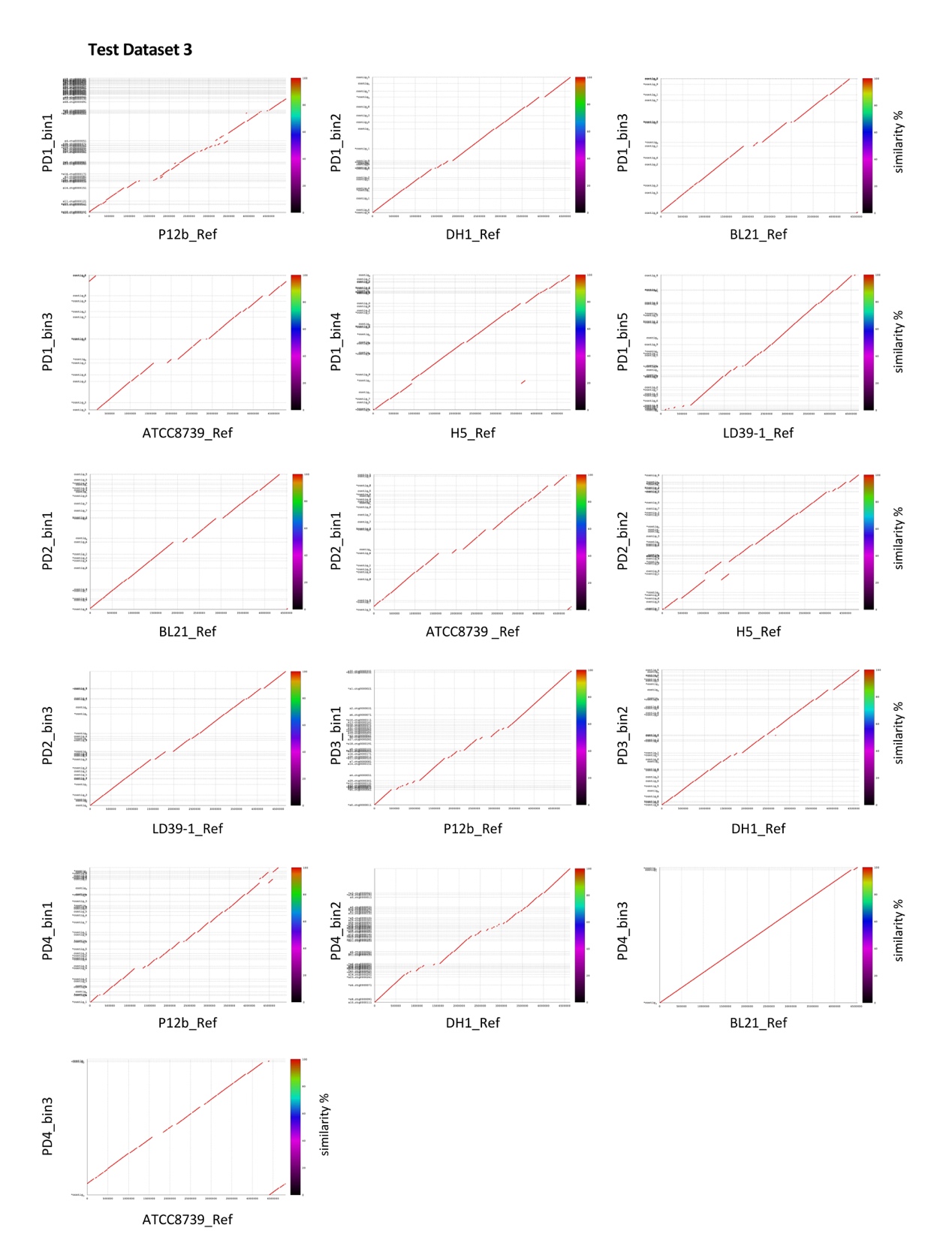


(Continued on the next page)


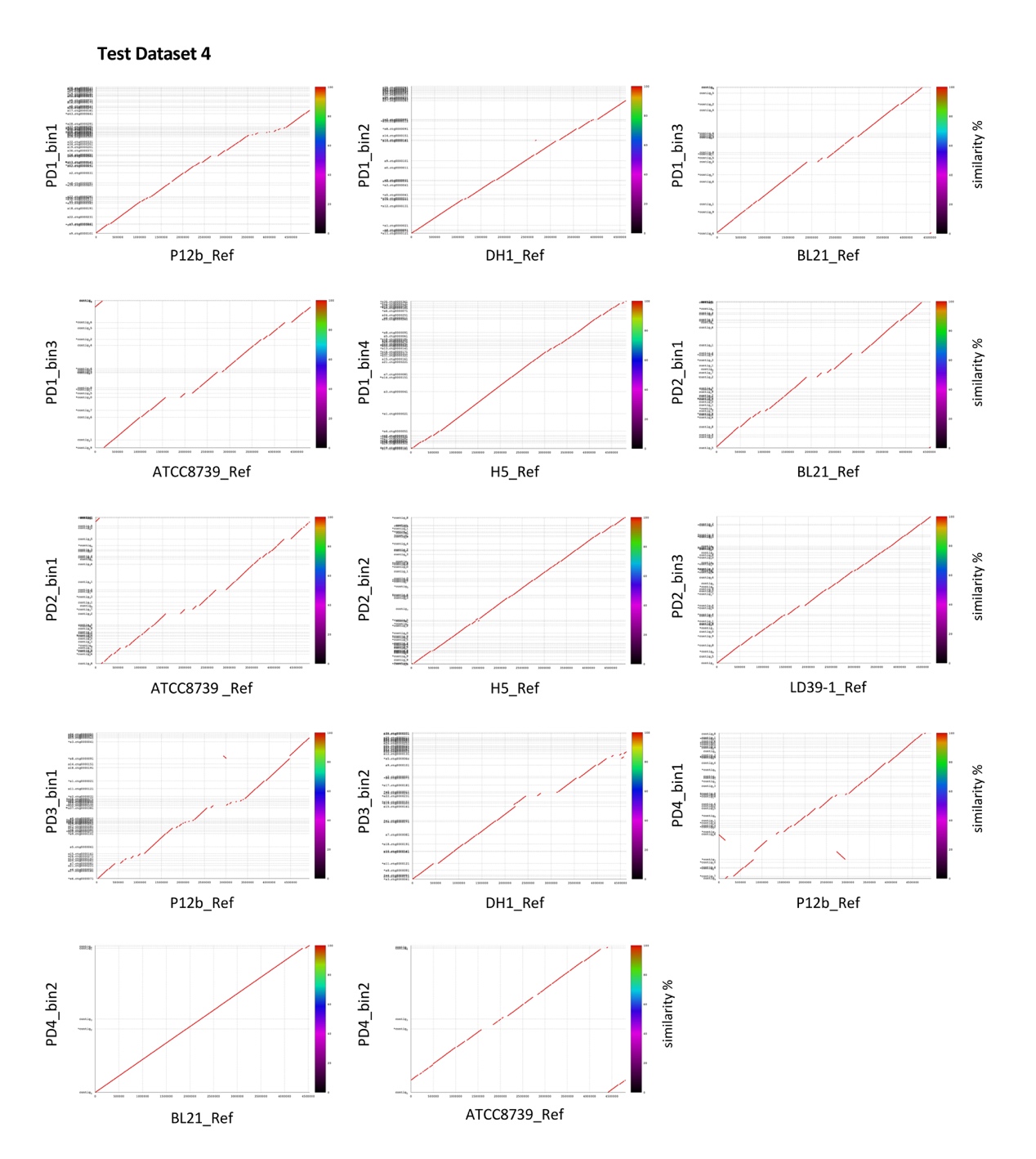


## Supplementary Figure 5: Synteny of the assembled bins obtained from *E. coli* test datasets 1 to 4 with their best hit reference genomes.


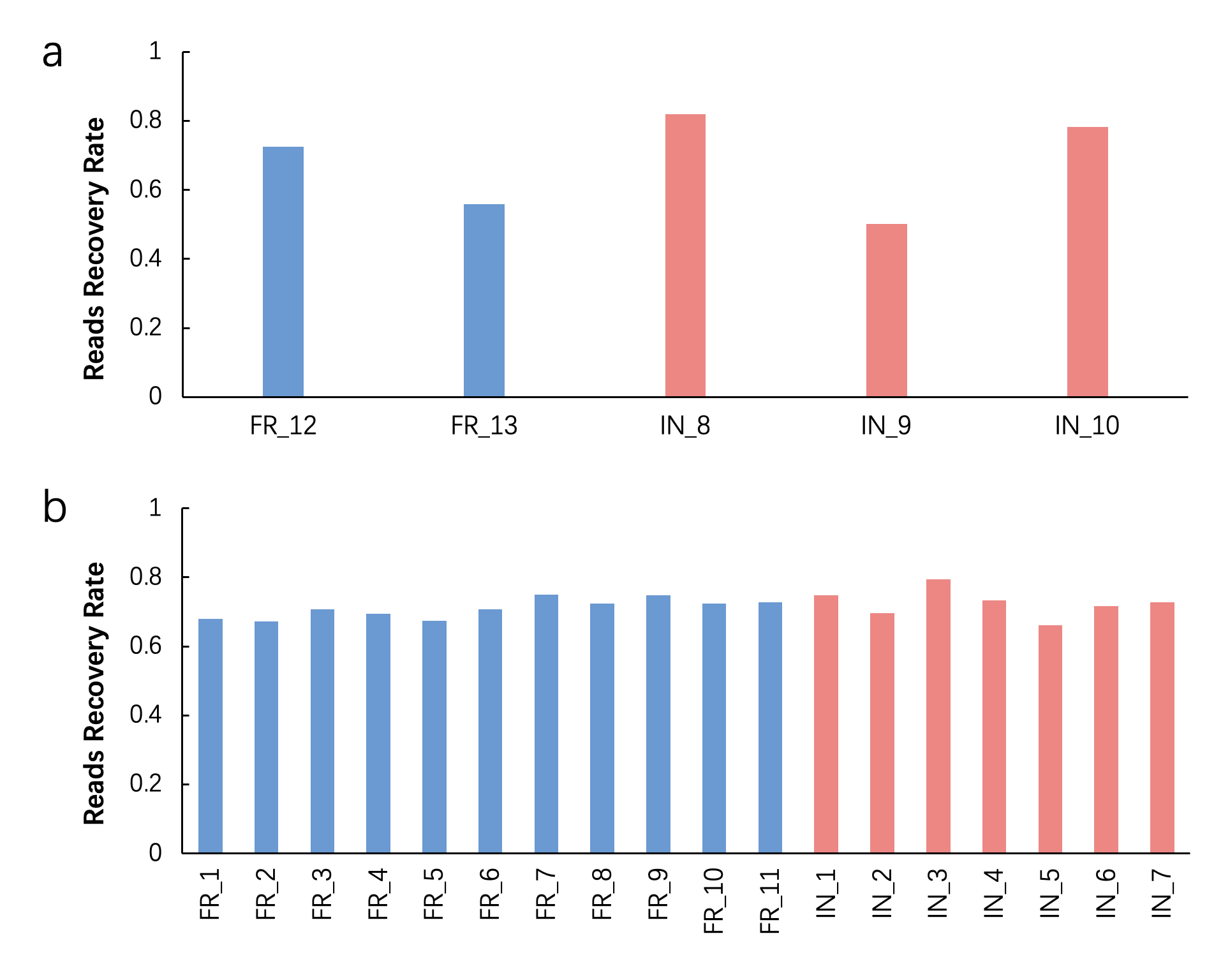


## Supplementary Figure 6: The reads recovery rate of PacBio (a) and Illumina (b) sequencing datasets.

Blue and red columns correspond to samples collected from methane seep (Formosa Ridge, FR) and hydrothermal vent (Iheya North Knoll, IN) sites.


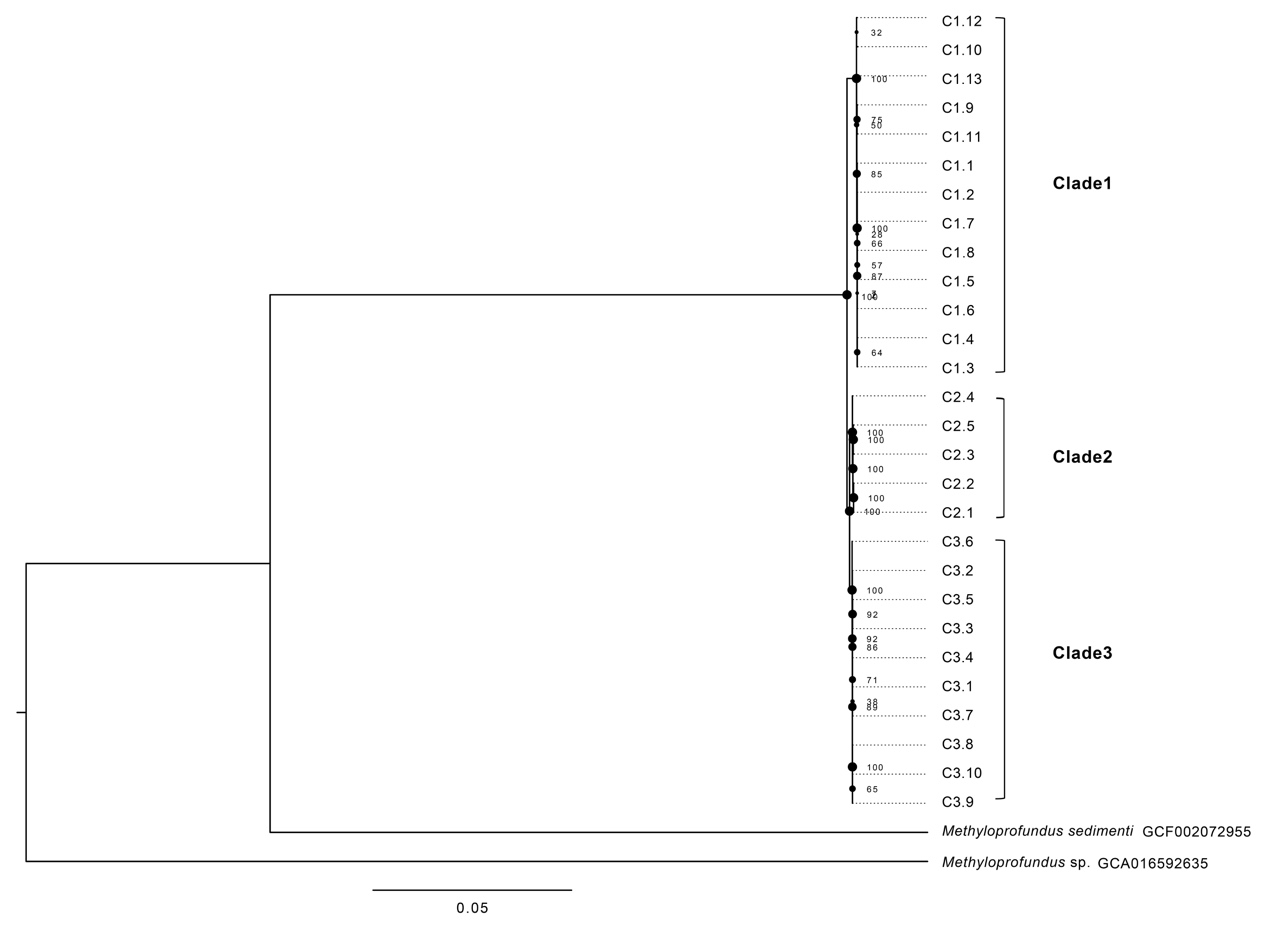


## Supplementary Figure 7: ML phylogenetic reconstruction of endosymbiont strains based on orthologous genes conserved across the pangenome.

*Methyloprofundus sedimenti* (GCF_002072955.1) and *Methyloprofundus* sp. (GCA_016592635.1) were used as outgroups in the analysis.


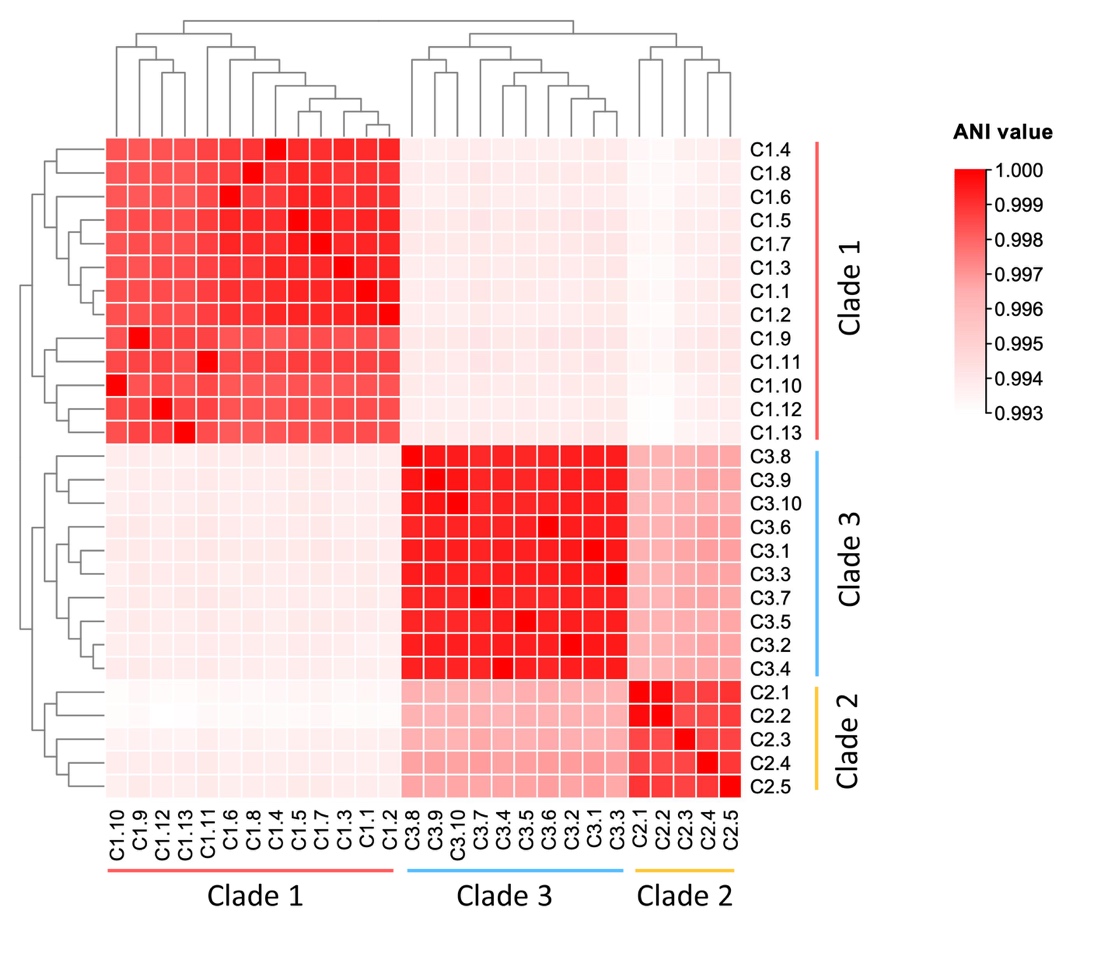


## Supplementary Figure 8: Heatmap of pairwise average nucleotide identities (ANI) of genome assemblies in the three clades.


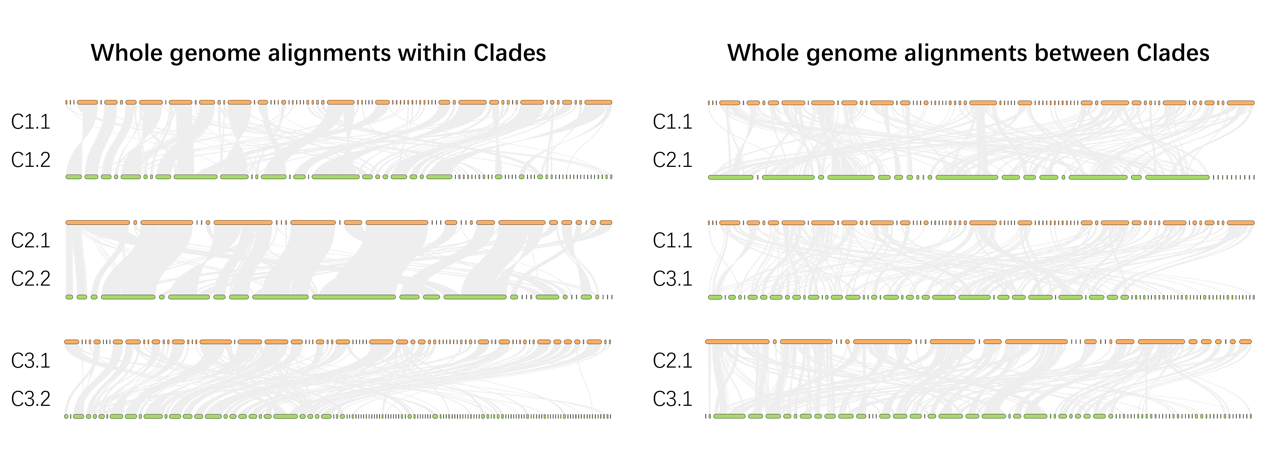


## Supplementary Figure 9: Whole genome alignments of representative endosymbiotic genomes in the three clades.

The representatives of a clade are the genome assemblies with the highest contig N50 value in each clade. C1.1 and C1.2 are the representative genomes in clade 1; C2.1 and C2.2 are the representative genomes in clade 2; C3.1 and C3.2 are the representative genomes in clade 3. Genome structure analysis through whole-genome alignments of representatives supported the differentiation among the three clades as indicated by phylogenetic analysis. The results revealed that genomes within the same clade were highly syntenic, with fewer rearrangements, insertions, or deletions, whereas genomes in different clades were far more structurally dynamic.


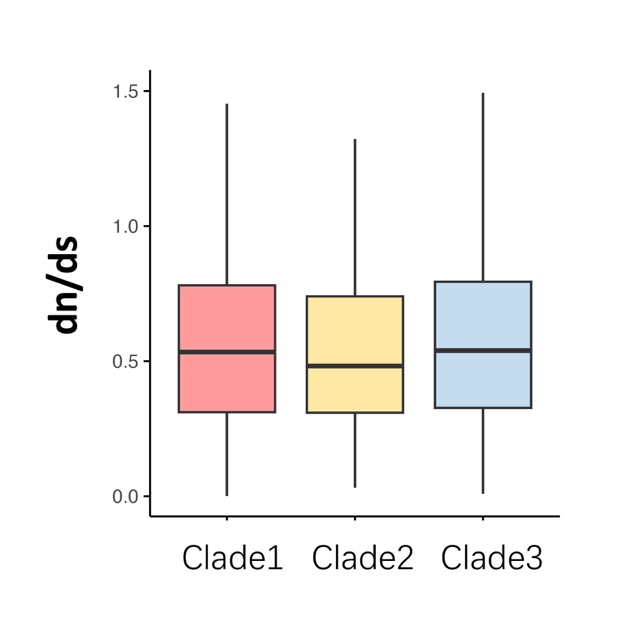


## Supplementary Figure 10: Box plot of the dN/dS values for each clade obtained from each ortholog.


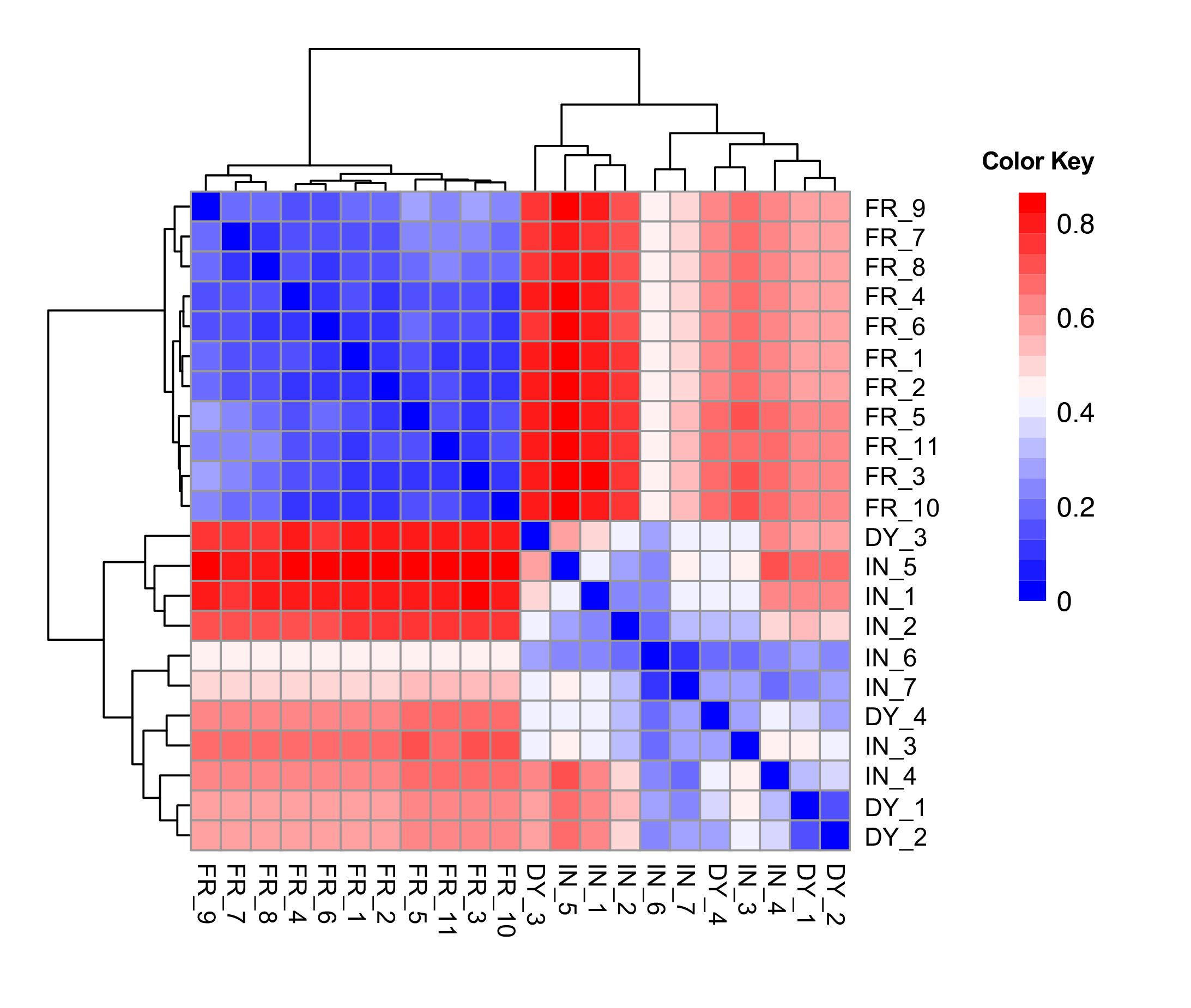


## Supplementary Figure 11: Heatmap showing the pairwise fixation index (*F*_ST_) value among individual mussels collected from the hydrothermal vents (Daiyon-Yonaguni Knoll, DY; Iheya North Knoll, IN) and the methane seep (Formosa Ridge, FR).


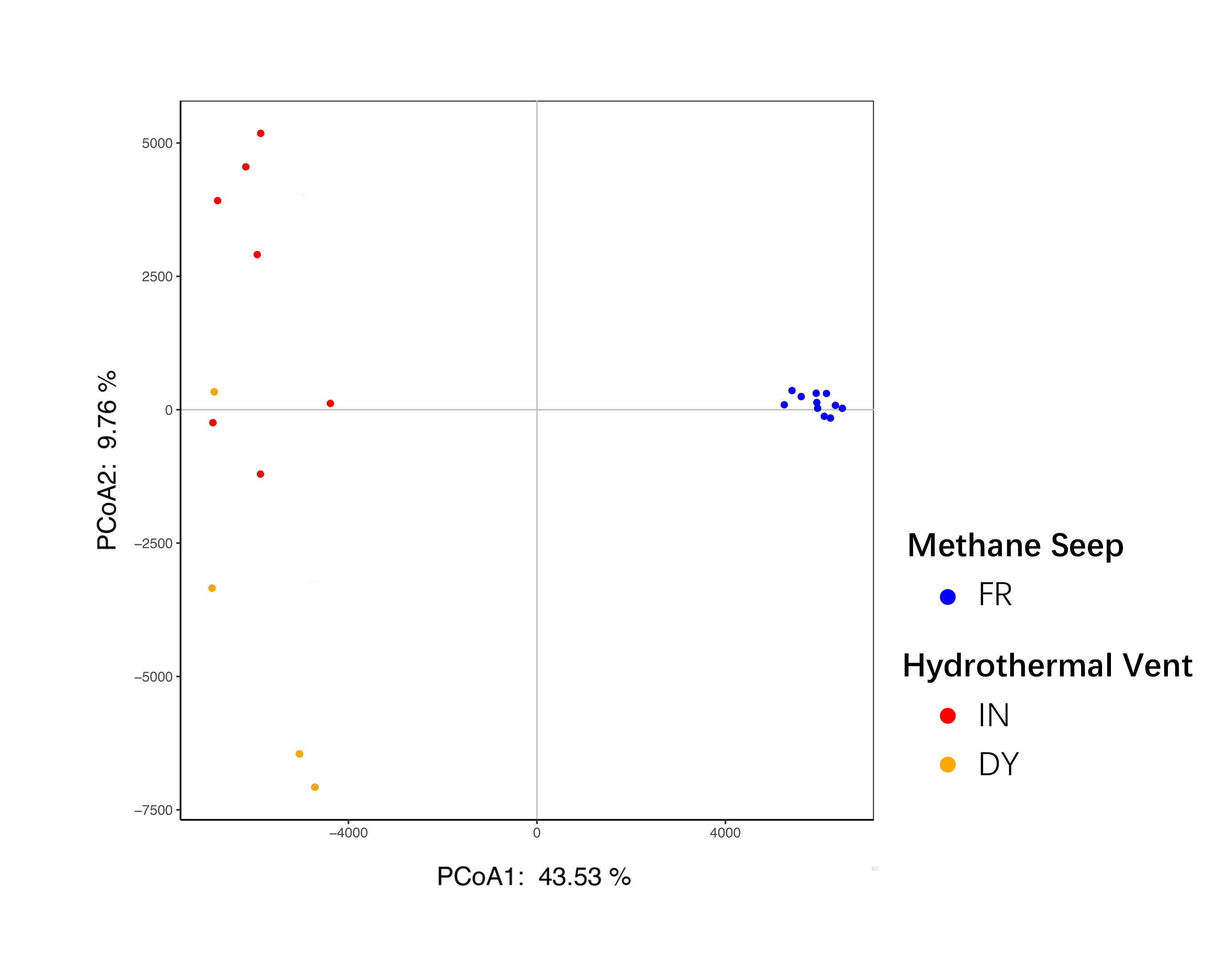


## Supplementary Figure 12: Principal coordinate analysis (PCoA) of individual mussels from the hydrothermal vents (Daiyon-Yonaguni Knoll, DY; Iheya North Knoll, IN) and the methane seep (Formosa Ridge, FR).

Colors correspond to mussel individuals collected from different sampling sites.


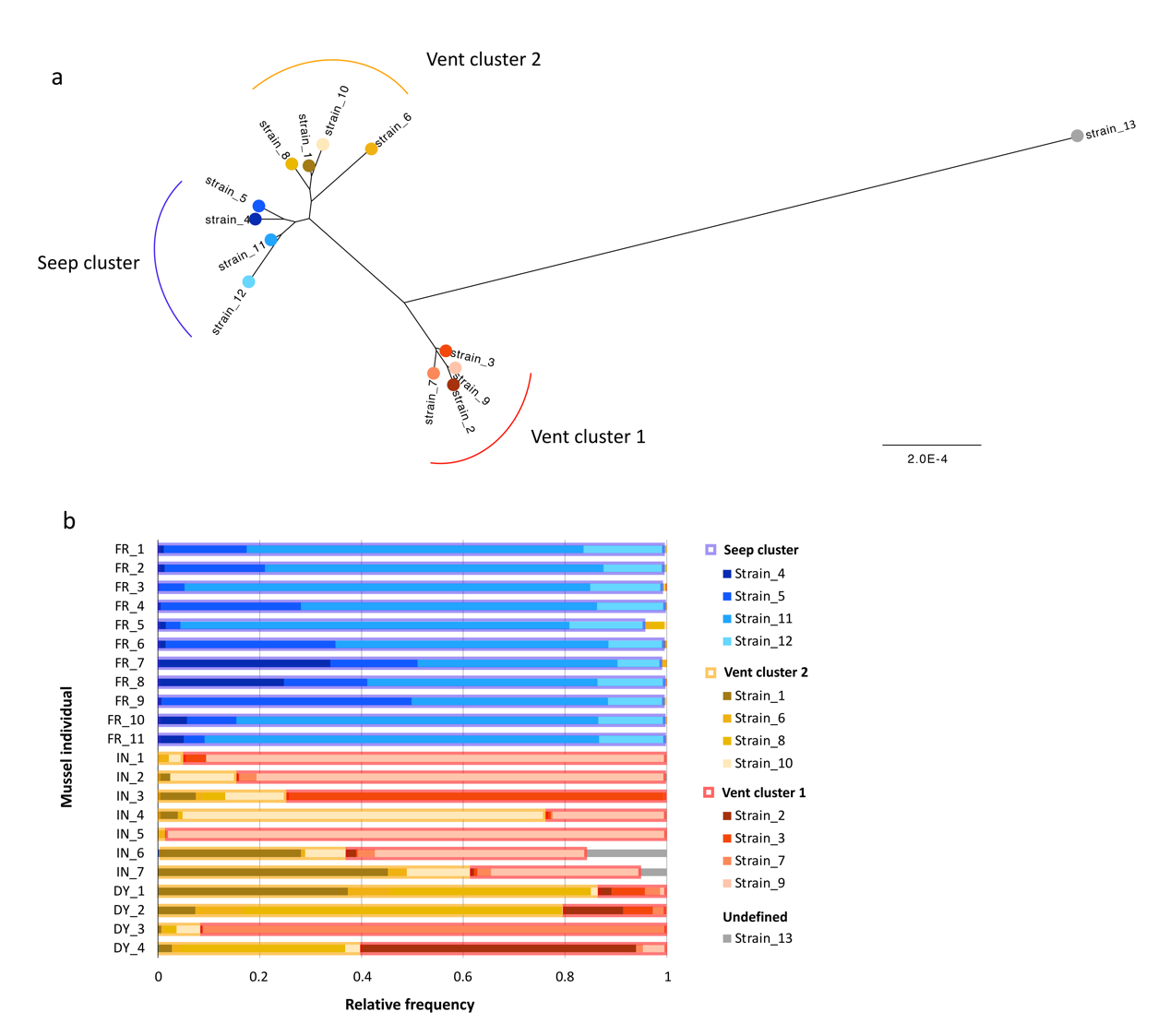


## Supplementary Figure 13: Phylogenetic relationship of symbiont strains reconstructed with DESMAN (a) and their relative abundance among individuals from the vent (Daiyon-Yonaguni Knoll, DY; Iheya North Knoll, IN) and seep (Formosa Ridge, FR) sites (b).


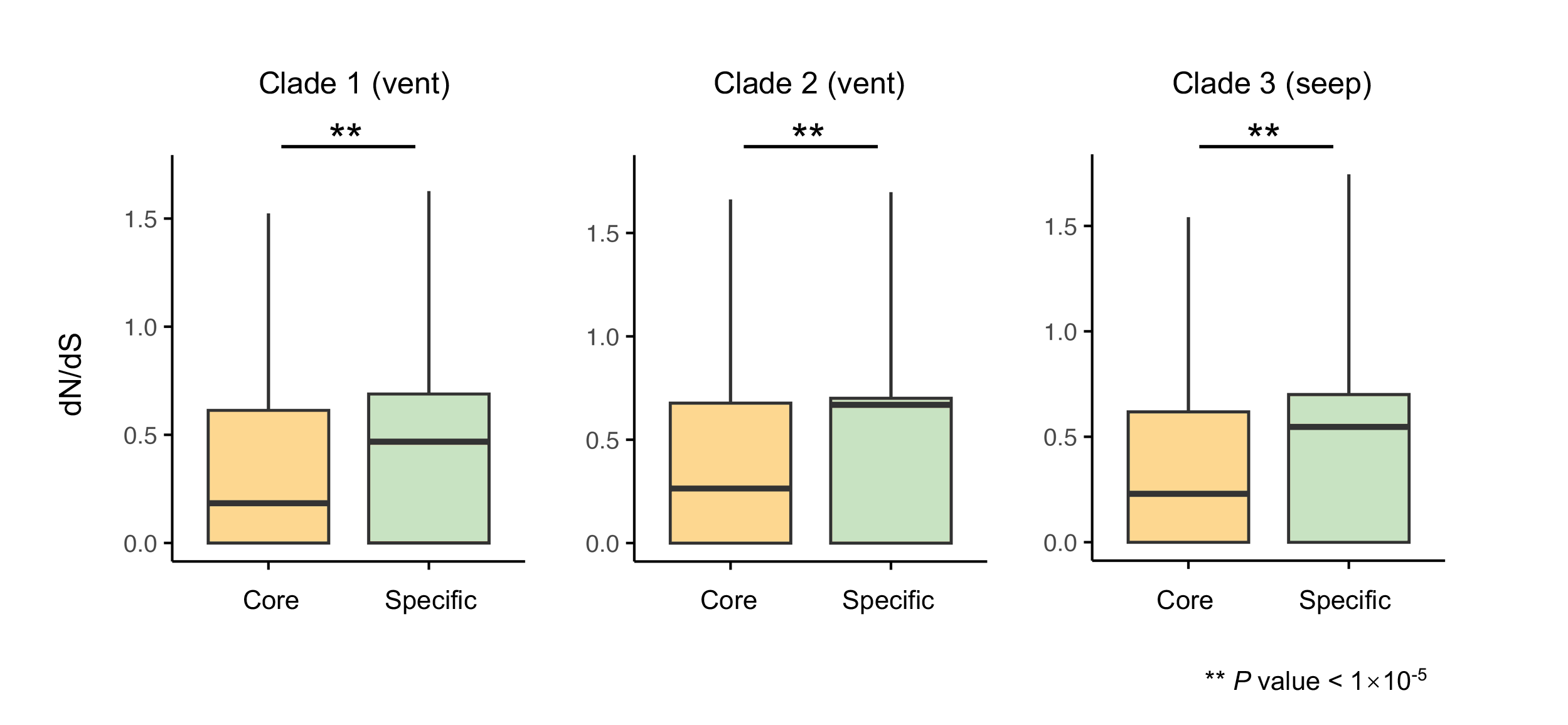


## Supplementary Figure 14: Box plot showing dN/dS values in the core genes and the clade-specific genes of the three clades.


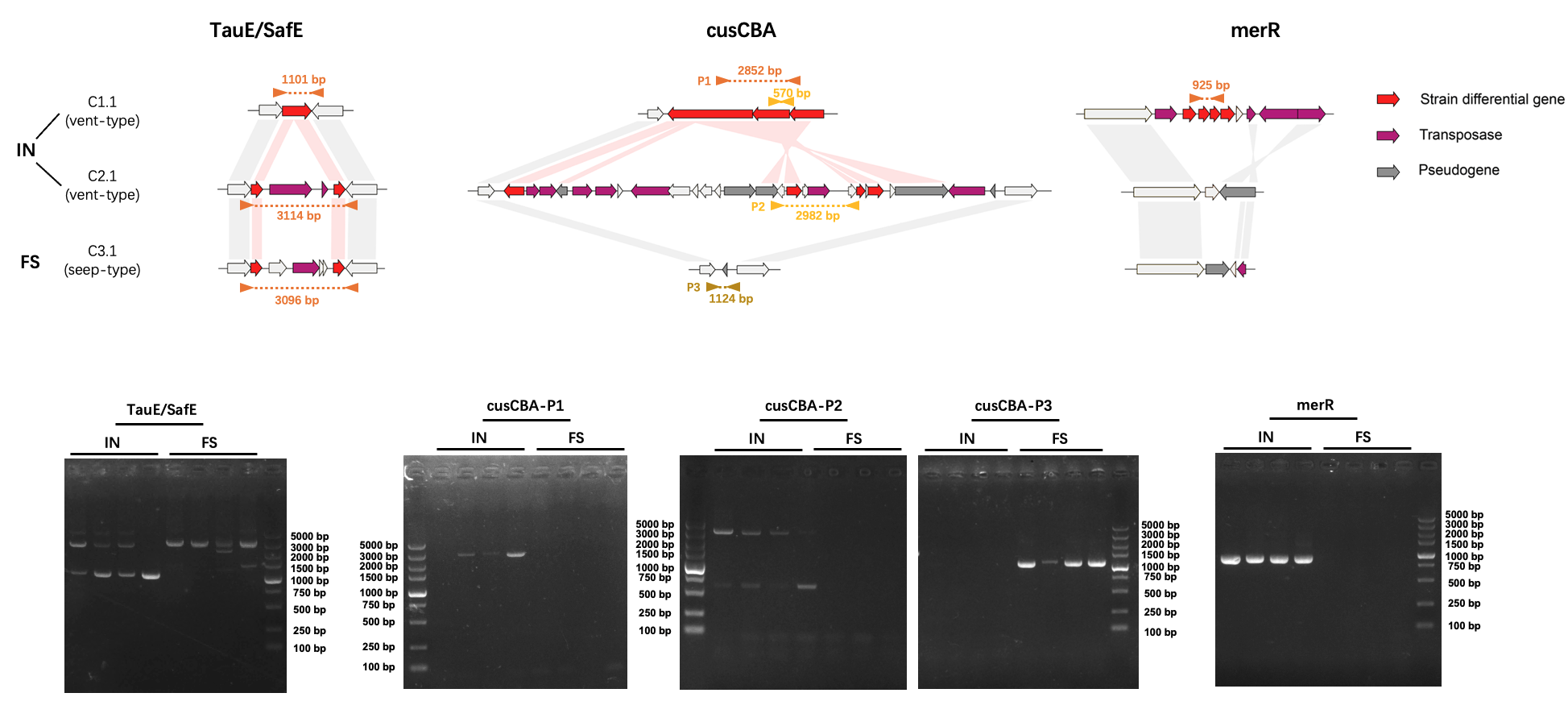


## Supplementary Figure 15: PCR amplification using gill DNA demonstrating the genomic variants among genomes from different clades.

Each of four individual mussels from the hydrothermal vent (Iheya North Knoll, IN) and the methane seep (Formosa Ridge, FR) were used for PCR amplification.


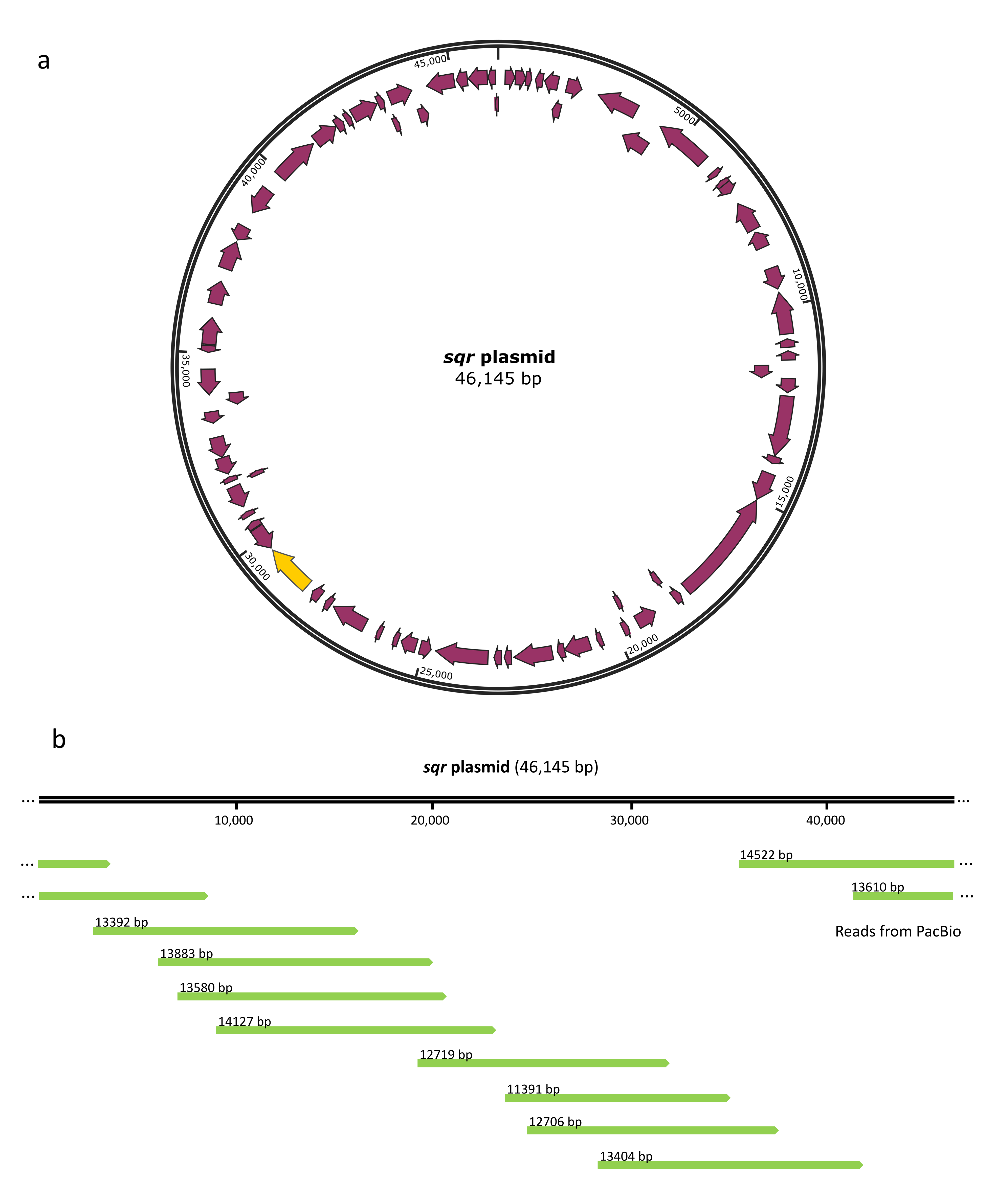


## Supplementary Figure 16: The assembled plasmid encoding sulfide:quinone oxidoreductase (*sqr*) gene.

(a) Genomic architecture of the circular *sqr* plasmid. The *sqr* gene is indicated by orange arrow. (b) Schematic diagram showing the reality of the *sqr* plasmid supported by PacBio sequencing reads. Green arrows indicate PacBio reads.

# Supplementary References

1. Gruber-Vodicka HR, Seah BKB, Pruesse E: phyloFlash: Rapid Small-Subunit rRNA Profiling and Targeted Assembly from Metagenomes. *mSystems* 2020, 5(5).

2. Hirayama H, Takaki Y, Abe M, Imachi H, Ikuta T, Miyazaki J, Tasumi E, Uematsu K, Tame A, Tsuda M *et al*: Multispecies Populations of Methanotrophic Methyloprofundus and Cultivation of a Likely Dominant Species from the Iheya North Deep-Sea Hydrothermal Field. *Appl Environ Microbiol* 2022, 88(2):e0075821.

3. Assie A, Borowski C, van der Heijden K, Raggi L, Geier B, Leisch N, Schimak MP, Dubilier N, Petersen JM: A specific and widespread association between deep-sea Bathymodiolus mussels and a novel family of Epsilonproteobacteria. *Environ Microbiol Rep* 2016, 8(5):805-813.

4. Assie A, Leisch N, Meier DV, Gruber-Vodicka H, Tegetmeyer HE, Meyerdierks A, Kleiner M, Hinzke T, Joye S, Saxton M *et al*: Horizontal acquisition of a patchwork Calvin cycle by symbiotic and free-living Campylobacterota (formerly Epsilonproteobacteria). *ISME J* 2020, 14(1):104-122.

5. Sun Y, Wang M, Zhong Z, Chen H, Wang H, Zhou L, Cao L, Fu L, Zhang H, Lian C *et al*: Adaption to hydrogen sulfide-rich environments: Strategies for active detoxification in deep-sea symbiotic mussels, Gigantidas platifrons. *Sci Total Environ* 2022, 804:150054.

6. Quince C, Delmont TO, Raguideau S, Alneberg J, Darling AE, Collins G, Eren AM: DESMAN: a new tool for de novo extraction of strains from metagenomes. *Genome Biol* 2017, 18(1):181.

7. Uritskiy GV, DiRuggiero J, Taylor J: MetaWRAP-a flexible pipeline for genome-resolved metagenomic data analysis. *Microbiome* 2018, 6(1):158.

8. Kang DD, Li F, Kirton E, Thomas A, Egan R, An H, Wang Z: MetaBAT 2: an adaptive binning algorithm for robust and efficient genome reconstruction from metagenome assemblies. *PeerJ* 2019, 7:e7359.

9. Alneberg J, Bjarnason BS, de Bruijn I, Schirmer M, Quick J, Ijaz UZ, Lahti L, Loman NJ, Andersson AF, Quince C: Binning metagenomic contigs by coverage and composition. *Nat Methods* 2014, 11(11):1144-1146.

10. Wu YW, Simmons BA, Singer SW: MaxBin 2.0: an automated binning algorithm to recover genomes from multiple metagenomic datasets. *Bioinformatics* 2016, 32(4):605-607.

11. Wang Z, Wang Z, Lu YY, Sun F, Zhu S: SolidBin: improving metagenome binning with semi-supervised normalized cut. *Bioinformatics* 2019, 35(21):4229-4238.

12. Li H: Minimap2: pairwise alignment for nucleotide sequences. *Bioinformatics* 2018, 34(18):3094-3100.

13. Liu J, Huang C, Shin DH, Yokota H, Jancarik J, Kim JS, Adams PD, Kim R, Kim SH: Crystal structure of a heat-inducible transcriptional repressor HrcA from Thermotoga maritima: structural insight into DNA binding and dimerization. *J Mol Biol* 2005, 350(5):987-996.

14. Jackowski S, Zhang YM, Price AC, White SW, Rock CO: A missense mutation in the fabB (beta-ketoacyl-acyl carrier protein synthase I) gene confers tiolactomycin resistance to Escherichia coli. *Antimicrob Agents Chemother* 2002, 46(5):1246-1252.

15. Ansorge R, Romano S, Sayavedra L, Porras MAG, Kupczok A, Tegetmeyer HE, Dubilier N, Petersen J: Functional diversity enables multiple symbiont strains to coexist in deep-sea mussels. *Nat Microbiol* 2019, 4(12):2487-2497.

16. Romero Picazo D, Dagan T, Ansorge R, Petersen JM, Dubilier N, Kupczok A: Horizontally transmitted symbiont populations in deep-sea mussels are genetically isolated. *ISME J* 2019, 13(12):2954-2968.

17. Boyd ES, Barkay T: The mercury resistance operon: from an origin in a geothermal environment to an efficient detoxification machine. *Front Microbiol* 2012, 3:349.

18. Freedman Z, Zhu C, Barkay T: Mercury resistance and mercuric reductase activities and expression among chemotrophic thermophilic Aquificae. *Appl Environ Microbiol* 2012, 78(18):6568-6575.

19. Chandrangsu P, Rensing C, Helmann JD: Metal homeostasis and resistance in bacteria. *Nat Rev Microbiol* 2017, 15(6):338-350.

20. Pal C, Asiani K, Arya S, Rensing C, Stekel DJ, Larsson DGJ, Hobman JL: Metal Resistance and Its Association With Antibiotic Resistance. *Adv Microb Physiol* 2017, 70:261-313.

21. Liu S, Dai J, Wei H, Li S, Wang P, Zhu T, Zhou J, Qiu D: Dissimilatory Nitrate Reduction to Ammonium (DNRA) and Denitrification Pathways Are Leveraged by Cyclic AMP Receptor Protein (CRP) Paralogues Based on Electron Donor/Acceptor Limitation in Shewanella loihica PV-4. *Appl Environ Microbiol* 2021, 87(2).

22. Jangir MM, Vani B, Chowdhury S: Analysis of seven putative Na(+)/H(+) antiporters of Arthrospira platensis NIES-39 using transcription profiling and in silico studies: an indication towards alkaline pH acclimation. *Physiol Mol Biol Plants* 2019, 25(5):1175-1183.

23. Krulwich TA, Sachs G, Padan E: Molecular aspects of bacterial pH sensing and homeostasis. *Nat Rev Microbiol* 2011, 9(5):330-343.

24. Avalos M, Garbeva P, Raaijmakers JM, van Wezel GP: Production of ammonia as a low-cost and long-distance antibiotic strategy by Streptomyces species. *ISME J* 2020, 14(2):569-583.

25. Slonczewski JL, Fujisawa M, Dopson M, Krulwich TA: Cytoplasmic pH Measurement and Homeostasis in Bacteria and Archaea. In*.*; 2009: 1-317.

26. McDowall JS, Murphy BJ, Haumann M, Palmer T, Armstrong FA, Sargent F: Bacterial formate hydrogenlyase complex. *Proc Natl Acad Sci U S A* 2014, 111(38):E3948-3956.

27. Lim JK, Mayer F, Kang SG, Muller V: Energy conservation by oxidation of formate to carbon dioxide and hydrogen via a sodium ion current in a hyperthermophilic archaeon. *Proc Natl Acad Sci U S A* 2014, 111(31):11497-11502.
